# Transcriptome regulation of carotenoids in five flesh-colored watermelons (*Citrullus lanatus*)

**DOI:** 10.1186/s12870-021-02965-z

**Published:** 2021-04-28

**Authors:** Pingli Yuan, Muhammad Jawad Umer, Nan He, Shengjie Zhao, Xuqiang Lu, Hongju Zhu, Chengsheng Gong, Weinan Diao, Haileslassie Gebremeskel, Hanhui Kuang, Wenge Liu

**Affiliations:** 1 Zhengzhou Fruit Research Institute, Chinese Academy of Agricultural Sciences, Zhengzhou, 450009 People’s Republic of China; 2 College of Horticulture and Forestry Sciences, Huazhong Agricultural University, Wuhan, 430070 People’s Republic of China

**Keywords:** *Citrullus lanatus*, Flesh color, Carotenoid, Transcriptional regulation, Candidate genes, WGCNA

## Abstract

**Background:**

Fruit flesh color in watermelon (*Citrullus lanatus*) is a great index for evaluating the appearance quality and a key contributor influencing consumers’ preferences. But the molecular mechanism of this intricate trait remains largely unknown. Here, the carotenoids and transcriptome dynamics during the fruit development of cultivated watermelon with five different flesh colors were analyzed.

**Results:**

A total of 13 carotenoids and 16,781 differentially expressed genes (DEGs), including 1295 transcription factors (TFs), were detected in five watermelon genotypes during the fruit development. The comprehensive accumulation patterns of carotenoids were closely related to flesh color. A number of potential structural genes and transcription factors were found to be associated with the carotenoid biosynthesis pathway using comparative transcriptome analysis. The differentially expressed genes were divided into six subclusters and distributed in different GO terms and metabolic pathways. Furthermore, we performed weighted gene co-expression network analysis and predicted the hub genes in six main modules determining carotenoid contents. *Cla018406* (a chaperone protein dnaJ-like protein) may be a candidate gene for β-carotene accumulation and highly expressed in orange flesh-colored fruit. *Cla007686* (a zinc finger CCCH domain-containing protein) was highly expressed in the red flesh-colored watermelon, maybe a key regulator of lycopene accumulation. *Cla003760* (membrane protein) and *Cla021635* (photosystem I reaction center subunit II) were predicted to be the hub genes and may play an essential role in yellow flesh formation.

**Conclusions:**

The composition and contents of carotenoids in five watermelon genotypes vary greatly. A series of candidate genes were revealed through combined analysis of metabolites and transcriptome. These results provide an important data resource for dissecting candidate genes and molecular basis governing flesh color formation in watermelon fruit.

**Supplementary Information:**

The online version contains supplementary material available at 10.1186/s12870-021-02965-z.

## Background

Watermelon (*Citrullus lanatus*) belongs to the Cucurbitaceae family and is originally cultivated in Africa. Now, watermelon has become one of the top five freshly consumed fruits, with China at the top in production and consumption of watermelon worldwide. Watermelon flesh contains many nutrients, such as lycopene, citrulline, and other health-promoting compounds related to the human diet [1]. Carotenoids are necessary for human life and health [2, 3]. Lycopene has been reported to be involved in the prevention of cancers and cardiovascular diseases [4]. The alpha-carotene, beta-cryptoxanthin, and beta-carotene are the main precursors of vitamin A [5], which plays an essential role in vision protection [6].

In plants, carotenoids are mainly involved in photosynthesis, light-harvest, and photoprotection [7]. Carotenoids are also the essential precursors of phytohormones (abscisic acid and strigolactones), which are key regulators for plant development and stress responses [8]. Apocarotenoids are carotenoids oxidative and enzymatic cleavage derivatives. Apocarotenoids participate in various biological processes of plant growth and development [9, 10], and also contribute to the flavor and aroma of flower petals or fruits [11].

Cultivated watermelons have the ability to synthesize various kinds of carotenoids in fruit, responsible for the vivid flesh colors, including white, yellow, orange, pink, red, and mixed color [12]. Watermelon is a suitable model species for studying the regulatory mechanisms of carotenoids biosynthesis in fleshy fruit owing to variously colored flesh. Lycopene is the main pigment in red-fleshed watermelons [13], xanthophylls (zeaxanthin and its derivatives, neoxanthin, and violaxanthin) are the main pigments in yellow-fleshed watermelons [14]. The β-carotene, ζ-carotene, prolycopene are the main pigments in orange-fleshed watermelons [15]. Some researches focus on the inheritance of flesh color in watermelon. The canary yellow (*C*) is dominant to red/pink/orange (*c*), the white flesh (*Wf*) is epistatic to the yellow flesh [16]. The *py* gene generated pale yellow flesh [17]. Scarlet red flesh, *Y*^*scr*^, is dominant to the coral red flesh [12]. Some quantitative trait loci (QTLs) associated with flesh color in watermelon have been reported. Two QTLs related to red flesh were identified on linkage groups 2 and 8 using an integrated genetic linkage map [18]. Bang et al. (2010) found a Clcyb.600 marker perfectly co-segregated with red or yellow flesh phenotypes [17]. The QTL related to the lycopene content and red flesh color was reported on chromosome 4 in a genetic population derived from red and pale-yellow flesh by Liu et al. (2015) [19]. The locus *Y*^*scr*^ was first mapped for the scarlet red flesh on chromosome 6 using a segregated population derived from scarlet- and coral- red flesh varieties [20]. The QTL associated with β-carotene accumulation in watermelon fruit was mapped on chromosome 1 [21]. The elevated chromoplast-localized phosphate transporter *ClPHT4;2* expression level is necessary for carotenoid accumulation and flesh color formation [22]. According to a recent study, the *ClLCYB* gene contributes to the red flesh color by decreasing its protein level instead of transcript level [23].

Some researches focused on the relationship of lycopene contents and gene expression level of lycopene metabolism spanning the period from young to mature fruits in watermelon [24, 25]. Comparative transcriptome analysis of red versus pale-yellow watermelons had been published by Zhu et al. (2017) [26]. However, the comprehensive molecular mechanisms underlying flesh color formation in various colored watermelon genotypes remain ambiguous, and rare regulators linked to watermelon flesh color have been reported on the basis of comparative transcriptome and co-expression network analysis. Here, we performed an integrated analysis of comparative transcriptome and carotenoids in five flesh-colored watermelons at different fruit development stages. Some candidate regulators were identified through pairwise transcriptome comparisons. The modules of co-expressed genes and hub genes for each carotenoid were confirmed by weighted gene co-expression network analysis (WGCNA). The data set provides a comprehensive view on the dynamic gene expression networks and their potential roles in controlling flesh color. This work also provides an important data basis for understanding the molecular regulation mechanism of watermelon flesh color formation.

## Results

### Flesh color assessment and carotenoids contents variation during fruit development of five watermelon genotypes

**Fig. 1:**
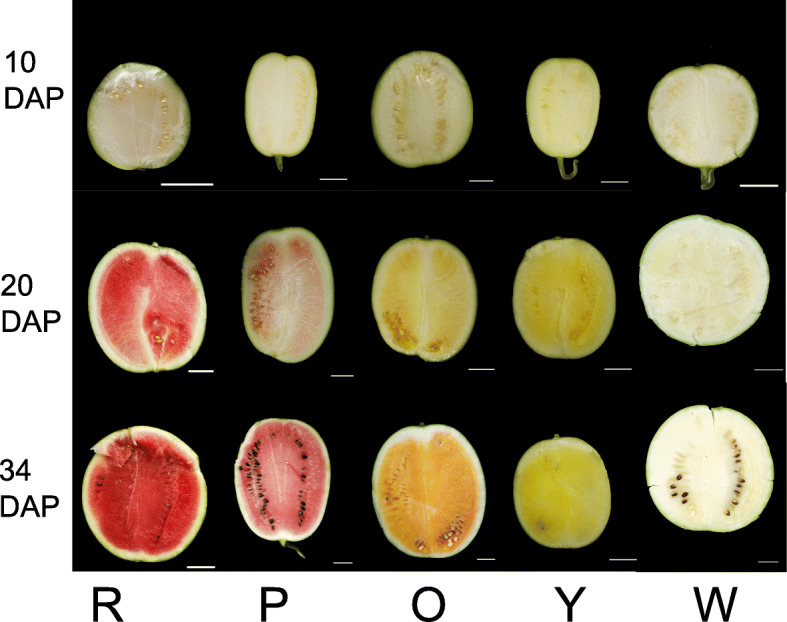
The flesh color of five different watermelon genotypes at 10 DAP, 20 DAP, and 34 DAP. R, P, O, Y, and W represents the red-, pink-, orange-, yellow-, and white-fleshed genotypes, respectively; DAP, days after pollination. Scale bars = 5 cm

Watermelon flesh features at different developmental stages have been shown in Fig. 1. We determined the color space values to confirm flesh color variations. At 10 days after pollination (DAP) all fruits were white flesh, there were no significant differences in color space parameters between different genotypes (Additional file 1: Table S1). At 20 DAP, the fruit’s flesh presents varying degrees of white, pink or yellow owe to carotenoids accumulation. The 20 DAP is a critical period for the rapid accumulation of pigments. At 34 DAP, the fruits were matured and the flesh has vivid colors except for the white flesh genotype. Significant differences of L*, a*, b*, and Chroma (C) were observed in different flesh-colored fruits at 20 DAP and 34 DAP. The differences in flesh color appeared at the 20 DAP and were more pronounced at 34 DAP in this study.

**Fig. 2:**
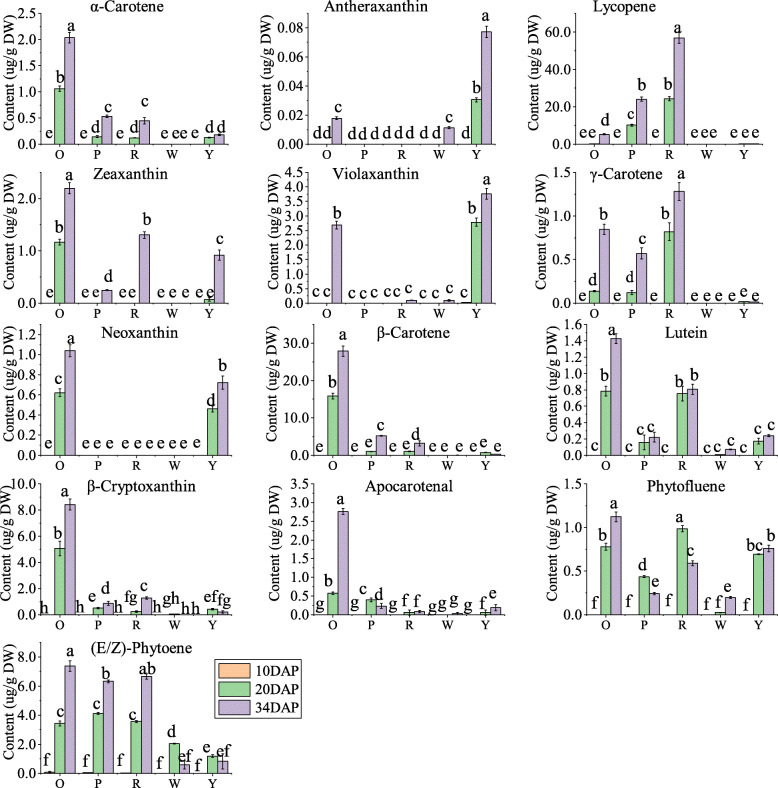
Contents of 13 metabolites of the carotenoid pathway. R, P, O, Y, and W represents the red-, pink-, orange-, yellow-, and white-fleshed genotypes, respectively; DAP, days after pollination; Data are shown as the means ± SD, *n* = 3. The different letters indicate the significant difference at 5% level according to Tukey’s post-hoc test

Flesh color in watermelon fruits is determined by carotenoids composition and contents. The carotenoids change in different fruits at three stages were measured using liquid chromatography-mass spectrometry (Additional file 1: Table S2, Fig. 2). Eighteen carotenoid standards were used and thirteen carotenoids were detected in this study. The PCA results and color comparison analysis suggested the reliability of metabolic data (Additional file 2: Fig. S1). At 10 DAP, no pigments were detected except for the trace amounts of phytoene in the young fruits. At 20 DAP and 34 DAP, the carotenoids contents gradually increased with the fruit development and ripening. The highest levels of lycopene, β-carotene, and violaxanthin were noted in the red-, orange-, and yellow-fleshed fruits, respectively. Lycopene was also the major pigments in pink-fleshed fruits. The carotenoids contents in white-fleshed fruit were very low, trace levels of phytofluene, phytoene, violaxanthin, antheraxanthin, and lutein were measured at 34 DAP (Additional file 1: Table S2, Fig. 2). Furthermore, the red flesh watermelon has the highest total carotenoids contents at 34 DAP, followed by orange, pink, yellow, and white flesh (Additional file 1: Table S2).

Taken together, the color space values and carotenoids levels revealed substantial variations among red, pink, orange, yellow, and white genotypes. It is conceivable that the DEGs at the three stages among 5 different flesh-colored watermelon fruits may play important roles in determining flesh color formation.

### Transcript sequencing of watermelon flesh with different colors

To explore the potential molecular mechanisms underlying the flesh coloration during the fruit development of 5 watermelon genotypes, RNA-Sequencing analysis was conducted on fruit flesh to generate transcriptome profiles. Samples of fruit flesh at three critical stages (10 DAP, 20 DAP, and 34 DAP) were obtained from five genotypes (Fig. 1). All samples were analyzed as three independent biological replicates.

**Fig. 3:**
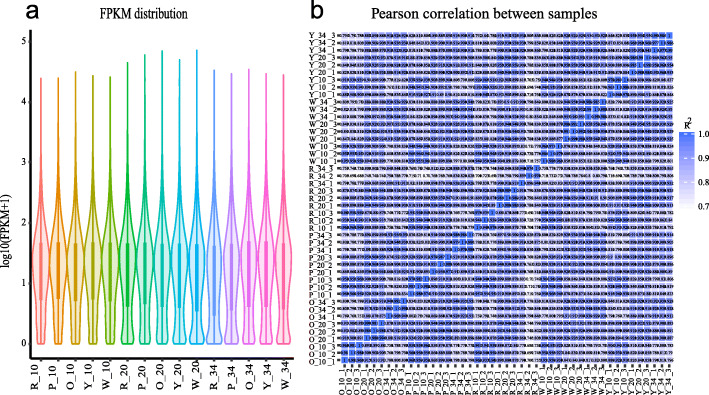
An overview of the transcriptome sequencing. Comparison of gene expression levels among different experimental groups (**a**). Pearson correlation analysis based on global RNA-seq data from 45 libraries (**b**). R, P, O, Y, and W represents the red-, pink-, orange-, yellow-, and white-fleshed genotypes, respectively

In total, 45 libraries were constructed and analyzed. After removing low quality reads, the average reads number per library were 51.9 million, with an average GC content of 44.15% (Additional file 1: Table S3). The RNA-Seq reads were aligned with the reference map of watermelon (97103) genome (http://cucurbitgenomics.org/organism/1) using HISAT (version 2.0.4) [27]. More than 97% of the total clean reads had Phred-like quality scores at the Q20 level (Additional file 1: Table S3). Ultimately, 24,794 genes (including 1354 novel genes) were identified by Cufflinks v2.1.1 [28]. The numbers of transcripts identified in each sample were expressed in FPKMs. Approximately 39.08% of expressed genes were in the 0–1 FPKM range, and 13.48% of expressed genes showed high expression levels (above 60 FPKM) (Additional file 1: Table S4). Genes with normalized reads lower than 1 FPKM were removed from the subsequent analysis. The gene expression levels among different experimental groups were compared in Fig. 3a. The expression patterns among biologically repeated samples were highly consistent (Fig. 3b) and the correlation coefficient was close to 1 (Additional file 1: Table S5). Therefore, this high-quality RNA-Seq data provided a solid foundation for identifying key genes participating in carotenoid synthesis during watermelon fruit development.

### Identification of differentially expressed genes in five genotypes

We conducted a pairwise comparison at three developmental stages of five genotypes to identify the genes correlating with watermelon flesh color. The DEGs were screened with FDR < 0.05, |log_2_(FoldChange)| > 1 as a threshold, the numbers and lists of significantly DEGs (up-regulated and down-regulated) of each pairwise comparison were shown in Additional file 1: Table S6 and Additional file 3-6: Dataset 1–4. Altogether, 16,781 genes were differentially expressed between at least one comparison.

**Fig. 4:**
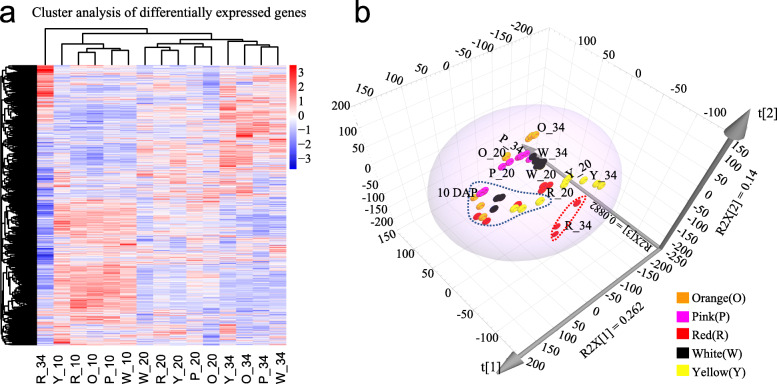
Hierarchical clustering analysis (**a**) and Principal component analysis (**b**) of the overall differentially expressed genes. The log10 (FPKM+ 1) value was normalized and transformed. R, P, O, Y, and W represents the red-, pink-, orange-, yellow-, and white-fleshed genotypes, respectively

The global hierarchical clustering (Fig. 4a) and principal component analysis (Fig. 4b) were performed based on the FPKM values for all DEGs. The results revealed that 45 samples could be generally assigned into three main groups corresponding to development stages based on gene expression patterns. The samples from 10 DAP were distinctly clustered as one group, the samples from 20 DAP and 34 DAP were clustered as another group except for the samples at 34 DAP of red flesh (Fig. 4a), suggesting that the expression patterns of most DEGs in different genotypes were consistent during fruit development. In particular, the differences in gene expression patterns between genotypes became clearer at 34 DAP as compared to that of 10 DAP and 20 DAP in the PCA analysis (Fig. 4b). At 20 DAP and 34 DAP, the white, pink, and orange genotypes were clustered together, while the yellow and red genotypes were separated from each other (Fig. 4b). Three biological repeats for red color at 34 DAP were not clustered together (Fig. 4b), which may be caused by environmental differences during cultivation. The difference in the overall gene expression pattern indicates that there must be a set of differentially expressed genes associated with the difference of flesh coloration in watermelon.

**Fig. 5:**
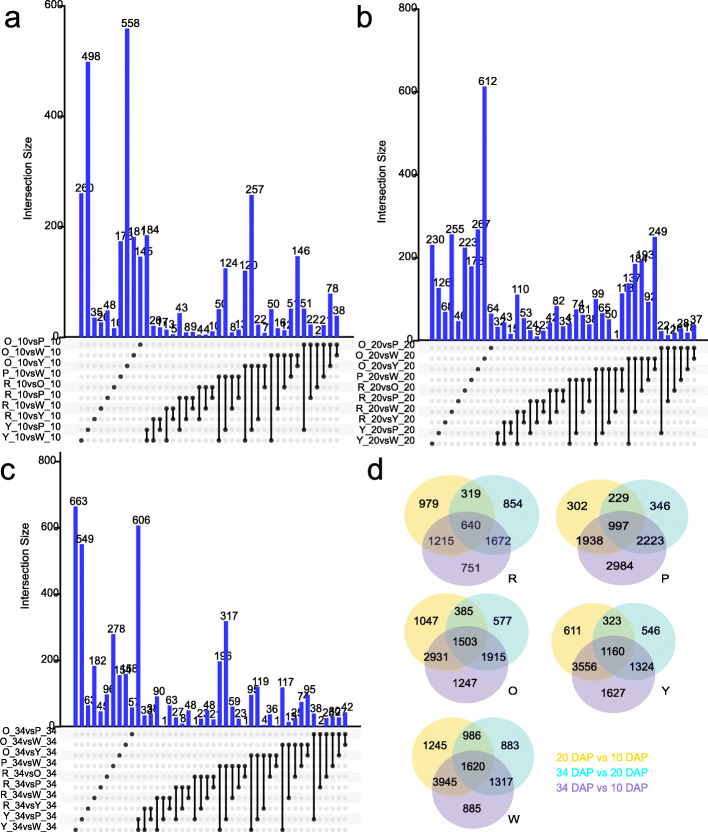
Venn diagrams of differentially expressed transcripts between 5 genotypes at 10 DAP(**a**), 20 DAP(**b**),34 DAP(**c**), and between the 3 stages of each genotype (**d**). R, P, O, Y, and W represents the red-, pink-, orange-, yellow-, and white-fleshed genotypes, respectively

At early developmental stage (10 DAP), a total of 5318 significantly differentially expressed genes were identified (Fig. 5a, Additional file 3: Dataset 1). Specifically, 510, 262, 588, 349 differentially expressed genes were identified in the red flesh genotype as compared to the pink, orange, yellow, and white flesh genotypes. The numbers of other pairwise comparisons were listed in Additional file 1: Table S6. The candidate gene linked to fruit shape, *ClFS1* (*Cla011257*), was differentially expressed in different genotypes at 10 DAP, consistent with the previous research [29]. *Cla019403* encodes xyloglucan endotransglucosylase is related to plant cell growth [30] and highly expressed at this stage (Additional file 2: Fig. S2a, Additional file 1: Table S7). An auxin response factor (*ARF*, *Cla009800*), a growth-regulating factor 5 (*GRF*, *Cla006802*), an auxin-induced SAUR-like protein (*Cla016617*), were associated with the fruit development and expansion [31] and highly expressed at early developmental stages (Additional file 2: Fig. S2a, Additional file 1: Table S7). There were fewer DEGs at 10 DAP as compared to later stages of watermelon fruit development.

At the pigment accumulation stage (20 DAP), 11,814 significantly differentially expressed genes were identified (Fig. 5b, Additional file 4: Dataset 2). Specifically, 2498, 4830, 3123, 4876 differentially expressed genes were identified in the red flesh genotype as compared to the pink, orange, yellow, and white flesh genotypes. The numbers of other pairwise comparisons were listed in Additional file 1: Table S6. The geranylgeranyl pyrophosphate synthase (*Cla015797*)*,* phytoene synthase protein (*Cla005425*), phytoene desaturase (Cla010898), carotenoid isomerase (*Cla017593*)*,* lycopene cyclase (*Cla016840*), violaxanthin de-epoxidase-related protein (*Cla007759*), 9-cis-epoxycarotenoid dioxygenase (*Cla015245*) were involved in carotenoid biosynthesis and differentially expressed among 5 genotypes (Additional file 2: Fig. S2b, Additional file 1: Table S7). Two *AP2-EREBPs* (*Cla000701*, *Cla017389*) and two *bHLHs* (*Cla020193*, *Cla022119*) were differentially expressed in 5 genotypes (Additional file 2: Fig. S2b, Additional file 1: Table S7). The expression level of *Cla017389* was positively related to the contents of lycopene (Pearson’s r = 0.85) and γ-Carotene (r = 0.69) in 15 experimental groups. The expression level of *Cla020193* was negatively correlated with the contents of phytofluene (r = − 0.60) and phytoene (r = − 0.57), the expression level of *Cla022119* was negatively correlated with the contents of phytofluene (r = − 0.59) and phytoene (r = − 0.58). The *AP2* and *bHLHs* are color regulators in other crops [2, 32].

At the maturity stage (34 DAP), 10,779 significantly differentially expressed genes were identified (Fig. 5c, Additional file 5: Dataset 3). Specifically, 2097, 2572, 2429, 3316 differentially expressed genes were identified in red flesh genotype as compared to pink, orange, yellow, and white flesh genotypes. The numbers of other pairwise comparisons were listed in Additional file 1: Table S6. Most of the carotenoid pathway genes and many TFs are differentially expressed among 5 genotypes at this stage. The geranylgeranyl reductase (*Cla003139*, *Cla019109*), geranylgeranyl pyrophosphate synthase (*Cla015797*, *Cla020121*), phytoene synthase protein (*Cla005425*, *Cla009122*), phytoene desaturase (*Cla010898*), carotenoid isomerase *(Cla017593*, *Cla011810)*, lycopene cyclase (*Cla005011*, *Cla017416*, *Cla016840*), 9-cis-epoxycarotenoid dioxygenase (*Cla015245*, *Cla009779*, *Cla005404*, *Cla005453*, *Cla019578*), beta-carotene hydroxylase (*Cla011420*, *Cla006149*), zeta-carotene desaturase (*Cla003751*), zeaxanthin epoxidase (*Cla020214*), and many TFs (*AP2-EREBPs*, *MADSs*, *MYBs*, *G2-likes*, *NACs*, *AUXs*), were differentially expressed at 34 DAP (Additional file 2: Fig. S2c, Additional file 1: Table S7). *Cla015245* and *Cla005404* (9-cis-epoxycarotenoid dioxygenase) were highly expressed in white flesh may lead to the degradation of xanthophyll and colorless flesh. Still, more notably, *Cla015245* and *Cla005404* have the highest expression levels in the mature pink fruit (Additional file 2: Fig. S2c, Additional file 1: Table S7). The results indicate that different genotypes have different color formation mechanisms. *Cla017389* and *Cla015515* (*AP2-ERFBP*) were highly expressed in red and pink fruits, respectively. *Cla017389* and *Cla015515* were homologous to ethylene-responsive transcription factor *RAP2–2* (E-value: 1.4e-28 and 3.0e-82) involved in the regulation of carotenoid biosynthesis in *Arabidopsis thaliana* [33]. More importantly, there was a positive correlation between the expression level of *Cla017389* and the contents of lycopene (r = 0.85). The transcription factor *bHLH* is related to the carotenoid metabolism in tomato [34], papaya [35], and citrus [36]. In this study, the expressions of *Cla006599* and *Cla022119* (*bHLH*) decreased at 20 and 34 DAP as compared to 10 DAP, and their expression patterns were similar to *CpbHLH1/2* that regulates carotenoid biosynthesis in papaya [35] (Additional file 2: Fig. S2c, Additional file 1: Table S7). Further analysis showed that the expression level of *Cla006599* was negatively correlated with the contents of phytofluene (r = − 0.56) and phytoene (r = − 0.62). The expression level of *Cla022119* was also negatively related to the contents of phytofluene and phytoene. Here we also note that a zinc finger CCCH domain-containing protein (*Cla007686*) showed a significant increase in expression level (~ 3 times) in red fruit at the ripening stage than that of early stage, (Additional file 1: Table S7), and the expression level was significantly associated with the lycopene content in 15 sample groups (r = 0.81). Five MYB related genes (*Cla020633*, *Cla007790*, *Cla009263*, *Cla017995*, and *Cla019223*) were differentially expressed in 5 genotypes at 34 DAP (Additional file 1: Table S7). The content of phytoene was positively correlated to the gene expression levels of *Cla009263* (r = 0.67) and *Cla017995* (r = 0.62).

For each genotype, 6430, 9019, 9605, 9147, and 10,881 developmental DEGs involved in fruit development were obtained in red-, pink-, orange-, yellow-, and white-fleshed watermelon genotypes, respectively (Fig. 5d, Additional file 6: Dataset 4). These results indicate that a large number of genes were involved in the regulation of watermelon fruit development. More genes were differentially expressed in the white flesh watermelon, suggesting a very complicated regulatory network of gene expression in this genotype. We also identified some differentially expressed genes related to fruit development using comparative transcriptome analysis. A cytokinin dehydrogenase gene (*Cla022463*) highly expressed at 10 DAP (Additional file 1: Table S7), may contribute to the early fruit development. The pyrabactin resistance 1-like protein (*PYL8*) could involve in the plant growth and stress responses by mediating ABA signaling in *Arabidopsis *[37]. Here, we found the expressions of four abscisic acid receptors *PYL8* (*Cla004235*, *Cla004904*, *Cla015009*, *Cla021167*) were significantly different in 5 watermelon genotypes (Additional file 2: Fig. S2d, Additional file 1: Table S7). The gene expression level of *Cla004904* had a negative correlation with the contents of antheraxanthin (r = − 0.61), violaxanthin (r = − 0.59) and neoxanthin (r = − 0.58). This gene may be involved in carotenoids degradation and abscisic acid metabolism in fruit development and ripening.

### Clustering of DEGs into six groups based on gene expression patterns

**Fig. 6:**
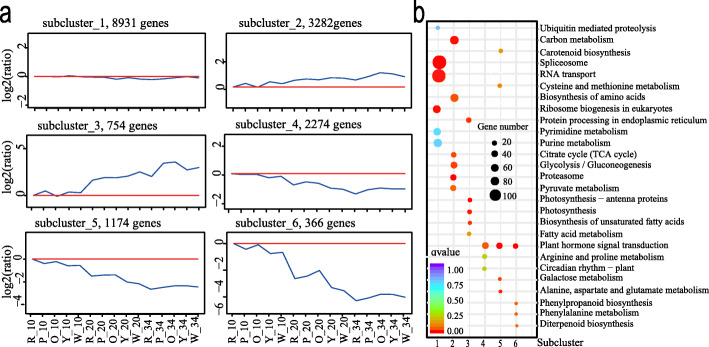
h-clustering of DEGs (**a**) and KEGG enrichment analysis (**b**). R, P, O, Y, and W represents the red-, pink-, orange-, yellow-, and white-fleshed genotypes, respectively

Based on the patterns of gene expression, the 16,781 DEGs were grouped into 6 different subclusters using h-cluster clustering approach (Fig. 6a, Additional file 1: Table S8). The genes grouped in the same cluster shared a similar expression pattern and have a similar function or participate in the same biological processes. Genes with relatively stable expression levels across different stages and genotypes were in subcluster 1. Genes expression exhibited a general downward trend in subclusters 2 and 3, whereas a general upward trend in subclusters 4, 5, and 6 according to the developmental stages (Fig. 6a).

To characterize the biological roles of DEGs in subcluster 1, GO enrichment analyses were performed. Almost the same proportion of genes were enriched into biological process, cellular component, and molecular function. GO terms were related to various basic life activities, such as binding, cellular macromolecule metabolic process, intracellular, and cell (Additional file 2: Fig. S3a, Additional file 7: Dataset 5). In addition, these DEGs were enriched to the spliceosome, RNA transport, and ribosome biogenesis pathways by Kyoto Encyclopedia of Genes and Genomes (KEGG) analysis (Fig. 6b, Additional file 7: Dataset 5). Previous studies showed that HY5 (ZIP) is involved in chloroplast biogenesis in Arabidopsis and tomato [38, 39]. Here, the transcription factors *Cla016581*, *Cla017361*, *Cla002873*, and *Cla021184* (*ZIP*) were differentially expressed in different samples (Additional file 1: Table S7). The gene expression of *Cla002873* was positively correlated to the contents of γ-Carotene (r = 0.77) and lycopene (r = 0.85). The gene expression of *Cla017361* was also positively correlated to the contents of γ-Carotene (r = 0.66) and lycopene (r = 0.72). Two *GLK2* TFs (*Cla010265*, *Cla020369*), were differentially expressed in different samples (Additional file 1: Table S7). The gene expression of *Cla010265* was positively correlated to the contents of lycopene (r = 0.77) and total carotenoids (r = 0.74). The gene expression of *Cla020369* was also related to the content of lycopene (r = 0.74). The expression level of transcription factor *Cla010815* was highest in red flesh fruit at 20 DAP and 34 DAP (Additional file 1: Table S7) and related to the lycopene content (r = 0.55). *Cla010815* (*MADS*) was homologous to *SlMADS1*, which plays an important role in fruit ripening as a repressive modulator in tomato [40] (E-value: 3e-76, identity: 77.24%). The expression of *Cla009725* (*MADS*) was negatively correlated with the content of phytoene (r = − 0.86), γ-carotene (r = − 0.82), lycopene content (r = − 0.73), and total carotenoids (r = − 0.85). This TF was homologous to *CsMADS6*, which was coordinately expressed with fruit development and coloration in citrus [41] (E-value: 4e-108, identity: 68.62%). The zinc finger CCCH domain-containing protein (*Cla007686*) was also in subcluster 1.

In subcluster 2, there were 3282 genes having a slightly higher expression at 20 DAP and 34 DAP than 10 DAP (Fig. 6a). GO terms such as single-organism metabolic process, small molecular metabolic process, and organonitrogen compound metabolic process were enriched (Additional file 2: Fig. S3b, Additional file 7: Dataset 5). KEGG enrichment analysis showed that the most significantly enriched pathways were proteasome, biosynthesis of amino acids, carbon metabolism pathway, TCA-cycle, glycolysis/gluconeogenesis proteasome, and pyruvate metabolism pathway. The proteasome pathway contains 26S protease regulatory subunit genes and proteasome subunit type genes. Acetyl-CoA carboxylase biotin carboxylase, pyruvate kinase, and malate dehydrogenase were in the pyruvate metabolism pathway (Fig. 6b, Additional file 7: Dataset 5). The transcription factor *Cla000691* was homologous to *SlMADS1* that plays as a repressive modulator in tomato fruit ripening [40] (E-value: 2e-87, identity: 64.93%) and highly expressed in the pink-fleshed watermelon fruits at later development stages (Additional file 1: Table S7).

Subcluster 3 represented genes that were highly expressed at 34 DAP, and the range of change was more obvious than that of subcluster 2 (Fig. 6a). 754 DEGs in this cluster mainly allocated into molecular function and biological process according to GO term analysis, with 310 and 287 DEGs were classified into the metabolic process and catalytic activity, respectively (Additional file 2: Fig. S3c, Additional file 7: Dataset 5). Notably, these DEGs were involved in pathways associated with photosynthesis-antenna proteins biosynthesis, photosynthesis, protein processing in endoplasmic reticulum, and biosynthesis of unsaturated fatty acids (Fig. 6b, Additional file 7: Dataset 5). *Cla006149* and *Cla011420* (beta-carotene hydroxylase), *Cla009122* (phytoene synthase), and *Cla009779* (9-cis-epoxycarotenoid dioxygenase) were related to the carotenoid pathway and differentially expressed in 5 genotypes (Additional file 2: Figs. S2b and S2c, Additional file 1: Table S7). In subcluster 3, some differentially expressed transcription factors were found (Additional file 7: Dataset 5). Some *MYBs*, *AP2-ERFBPs*, *bHLHs*, *NACs*, and *WRKYs* may be important regulators in fruit development and ripening [2]. Two *MYBs* (*Cla018631* and *Cla006739*) and two *WRKY*s (*Cla002243* and *Cla002084*) were highly expressed in yellow and pink flesh, respectively (Additional file 2: Fig. S2e, Additional file 1: Table S7). The gene expression levels of *Cla018631* and *Cla006739* were correlated with the contents of antheraxanthin and violaxanthin (r: *Cla018631 –* antheraxanthin = 0.88, *Cla018631 -* violaxanthin = 0.69, *Cla006739 –* antheraxanthin = 0.85, *Cla006739 -* violaxanthin = 0.69). The gene expression of *Cla002243* was negatively correlated with the content of violaxanthin (r = − 0.58), but there was a positive correlation between the expression of *Cla002084* and the content of phytoene (r = 0.64).

There were 2274, 1174, and 366 genes in subclusters 4, 5, and 6, respectively. These genes were highly expressed at 10 DAP and decreased to low gene expression levels at the later stages with different change magnitudes (Fig. 6a). GO enrichment analysis of subcluster 4 indicated that biological processes were most enriched. (Additional file 2: Fig. S3d, Additional file 7: Dataset 5). KEGG analysis showed that genes were involved in plant hormone signal transduction pathways, such as signal transduction histidine kinase (*Cla000685*, *Cla005808*), auxin responsive protein (*Cla003635*), and Ein3-binding f-box protein (*Cla020970*) (Fig. 6b, Additional file 7: Dataset 5). The transcription factor *Cla019630* (*MADS*) in subcluster 4 was homologous to *CsMADS6* that coordinately expressed with citrus fruit development and coloration [41] (E-value: 2e-98; Identity: 75.27%), its gene expression was negatively related to content of violaxanthin (r = − 0.57). GO enrichment of subcluster 5 genes assigned to the biological process and molecular function, such as protein phosphorylation, protein kinase activity (Additional file 2: Fig. S3e, Additional file 7: Dataset 5). Genes were enriched into plant hormone signal transduction, alanine, aspartate, glutamate metabolism, and others by KEGG (Fig. 6b, Additional file 7: Dataset 5). In subcluster 6, the enriched GO terms were predominantly related to molecular function and biological processes, such as enzyme inhibitor activity and endopeptidase regulator activity (Additional file 2: Fig. S3f, Additional file 7: Dataset 5). KEGG enrichment was mostly related to the pathways of plant hormone signal transduction, phenylpropanoid biosynthesis, and phenylalanine metabolism (Fig. 6b, Additional file 7: Dataset 5). *Cla019806*, *Cla0004102*, *Cla002975*, and *Cla016617* were involved in hormone synthesis and highly expressed at early stages of fruit development as compared to later fruit developmental stages (Additional file 2: Fig. S2f, Additional file 7: Dataset 5, Additional file 1: Table S 7).

### Co-expression network analysis identified carotenoid-related DEG*s*

To identify the potential genes (structural genes and putative transcription factors) highly associated with different kinds of carotenoids accumulation. The carotenoids content in each sample was used as phenotypic data and 16,781 DEGs were used to perform the weighted gene co-expression network analysis (WGCNA).

**Fig. 7:**
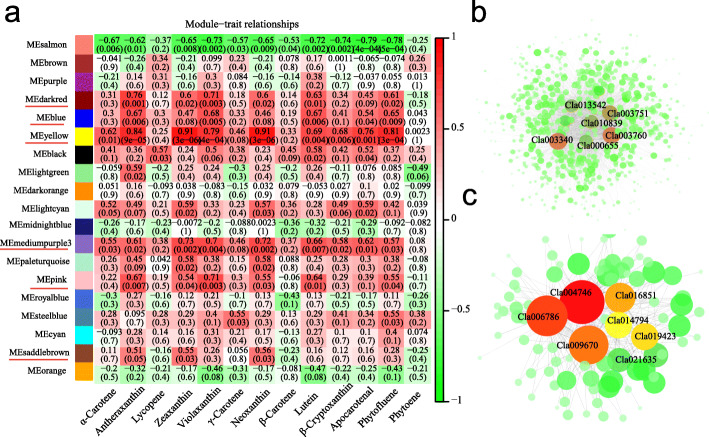
Module-carotenoid relationship (**a**) and correlation network analysis of yellow (**b**) and darkred (**c**) module. Each row corresponds to a module, labeled with color as in Additional file 2: Fig. S5a. The cell’s value at the row-column intersection indicates the correlation coefficient between the module and the carotenoid and is displayed according to the color scale on the right. The value in parentheses in each cell represents the *P*-value. Top 10 genes with a high degree of connectivity and their associated edges were displayed

A sample dendrogram and trait heatmap was constructed to illustrate the expression of each phenotypic parameter at different developmental stages **(**Additional file 2: Fig. S4a**)**. The best parameter value determination for module construction was 7.7 for this dataset **(**Additional file 2: Fig. S4b**)**. A total of 40 distinct co-expression modules were formed according to the pairwise correlations of gene expression across all samples and co-expression patterns of individual genes, as shown in the cluster dendrogram (Additional file 2: Fig. S5a). Moreover, a network heatmap of all the DEGs in gene-modules was also drawn to exhibit the correlation between modules (Additional file 2: Fig. S5b). Notably, six co-expression modules (indicated with red underlines) have a high positive correlation with most carotenoids (Fig. 7a), meaning that genes in these modules play an important role in carotenoids accumulation.

‘Yellow’ module contains 846 genes (including 34 TFs) (Additional file 1: Table S9) exhibited a stronger positive relationship with zeaxanthin (correlation coefficient, r = 0.91), neoxanthin(r = 0.91), antheraxanthin (r = 0.84), violaxanthin (r = 0.79), phytofluene (r = 0.81), apocarotenal (r = 0.76), *β*-cryptoxanthin (r = 0.68), lutein (r = 0.69), and α-carotene (r = 0.62) (Fig. 7a). In this module, a set of genes related to the cellular metabolic process and involved in pyruvate metabolism and proteasome pathway were identified (Additional file 2: Fig. S6). Pyruvate is an important mediator of carbohydrate, fat, and protein metabolism, and participates in several important metabolic pathways in vivo. The proteasome is related to the regulation of carotenoid content in tomato [42]. This module contains differentially expressed genes between the yellow−/orange-fleshed genotypes and the red−/pink−/white-fleshed genotypes (Additional file 2: Fig. S7a), and maybe the important factors involved in yellow pigments accumulation. According to gene function annotation, *Cla005011* was lycopene beta-cyclase in watermelon [23]. *Cla003751* encodes a zeta-carotene desaturase, *Cla020214* encodes zeaxanthin epoxidase. Hence, they were involved in the carotenoid pathway (Additional file 2: Fig. S2c, Additional file 1: Table S7 and S9). *Cla018406* (a chaperone protein dnaJ-like protein) was highly expressed in orange and yellow flesh and its gene expression level was related to the β-Carotene content (r = 0.71) and neoxanthin content (r = 0.93). The *Cla014416* (plastid-lipid-associated protein, *ClPAP*) was homologous to *SlPAP* (NP_001234183.1) that affect carotenoid content in tomato [43] (E-value: 1e-145, Identity: 68.67%). The expression level of *Cla014416* was higher in yellow−/orange-flesh than in red−/pink−/white-flesh genotypes used in this study (Additional file 2: Fig. S2g, Additional file 1: Table S7 and S9). This result was different from the previous report that *ClPAP* highly expressed in red/orange than yellow /white color genotypes [44], possibly due to different genotypes used in these two studies. *Cla000655* (encodes a cytochrome P450) was homologous to the protein lutein deficient 5 (*CYP97A3*), which was involved in the biosynthesis of xanthophylls in *Arabidopsis thaliana* [45] (E-value: 6.5e-267, Identity: 80.47%). *Cla010839* was homologous to 15-cis-zeta-carotenoid isomerase in *Arabidopsis thaliana* [46] (E-value: 1.1e-135, Identity: 67.43%). *Cla018347* (encodes a cytochrome P450) was related to carotenoid epsilon-monooxygenase (*CYP97C1*) in *Arabidopsis thaliana* [47] (E-value: 1.2e-233, Identity: 76.20%). *Cla000655*, *Cla010839*, and *Cla018347* were highly expressed in yellow and orange color fruits (Additional file 2: Fig. S2g, Additional file 1: Table S7 and S9) and maybe involved in the biosynthesis of xanthophylls in watermelon. The MYB transcription factor can regulate the carotenoid contents in *Mimulus lewisii* flowers [48]. *Cla013280* and *Cla010722* belong to the MYB family and were highly expressed in yellow-fleshed fruits (Additional file 2: Fig. S2g, Additional file 1: Table S7 and S9). The gene expression of *Cla013280* was positively related to the contents of antheraxanthin (r = 0.85) and violaxanthin (r = 0.81). The gene expression of *Cla010722* was also related to the contents of antheraxanthin (r = 0.75) and violaxanthin (r = 0.75). The hub genes linked to this module were further analyzed using Cytoscape cytoHubba (Fig. 7b), the ATP synthase protein I, (*Cla013542*), cysteine desulfuration protein SufE (*Cla003340*), membrane protein (*Cla003760*), and others were identified as hub genes responsible for yellow color formation in watermelon (Additional file 2: Fig. S8, Additional file 1: Table S9 and S10).

The ‘dark-red’ module containing 111 genes was positively associated with the contents of antheraxanthin and violaxanthin, having a correlation coefficient of 0.76 and 0.71 respectively. Heatmaps (Additional file 2: Fig. S7b) showed that the ‘dark-red’ module-specific genes were represented the samples (yellow, orange, white) rich in antheraxanthin and violaxanthin. *Cla004704* encodes a photosystem II oxygen evolving complex protein PsbP, *Cla005429* encodes an oxygen-evolving enhancer protein 2, chloroplastic, *PsbP*. *Cla004746* encodes a chlorophyll a-b binding protein 6A (Additional file 2: Fig. S2g, Additional file 1: Table S7 and S9). *Cla021635* encodes a photosystem I reaction center subunit II, rank as the top hub gene in this module (Fig. 7c, Additional file 2: Fig. S8, Additional file 1: Table S10). Many genes in this module were also related to chloroplast or photosystem I, II (Additional file 1: Table S7).

The ‘mediumpurple 3’ module, with 32 identified genes, was highly correlated to the contents of α-carotene, violaxanthin, neoxanthin, lutein, and zeaxanthin with the correlation coefficient of 0.55, 0.70, 0.72, 0.66, and 0.73, respectively (Fig. 7a). The heatmap was shown in Additional file 2: Fig. S7c. *Cla005637*, *Cla017046*, and *Cla011297* were identified as hub genes for this module (Additional file 2: Fig. S8, Additional file 1: Table S10). The ‘black’ module was specific to lycopene contents (r = 0.57) and lutein (r = 0.58). The ‘steelblue’ module was specific to the contents of γ-carotene (r = 0.55) and phytofluene (r = 0.55), respectively (Fig. 7a). The transcription factor *Cla019630* (*MADS*) gene mentioned above was also in the ‘saddlebrown’ module, which was correlated to the contents of zeaxanthin (r = 0.55), neoxanthin (r = 0.56), and antheraxanthin (r = 0.51) (Fig. 7a). Their hub genes were listed in Additional file 1: Table S10 and expression levels were shown in Additional file 2: Fig. S8.

By WGCNA, we found that most carotenoid pathway genes which were present in the yellow module (Additional file 2: Fig. S7a). Co-expression networks were drawn to identify hub genes linked to flesh carotenoids contents in eight of the selected modules (Additional file 1: Table S10).

### Validation of the expression of key DEGs by qRT-PCR

Twenty-one DEGs were used for qRT-PCR analysis to verify the quality of RNA-Seq data. We found a strong correlation between the RNA-Seq and qRT-PCR data (r = 0.90 ~ 0.99, the correlation was calculated separately for each gene), indicating the reliability of our transcriptome data (Additional file 2: Fig. S9).

## Discussion

### Carotenoids in different flesh-colored watermelons

Carotenoids are the second most abundant natural pigments worldwide [3], that widely exists in fruits, vegetables, and flowers. Carotenoids are divided into two subgroups, namely, carotenes (non-oxygenated, β-carotene, lycopene, α-carotene, δ-carotene, γ-carotene, 15-cis-phytoene, and 9,15,9′-tri-cis-ζ-carotene) and xanthophylls (oxygenated, lutein, violaxanthin, α-cryptoxanthin, β-cryptoxanthin, zeinoxanthin, zeaxanthin, violaxanthin, and neoxanthin) [2]. The different compositions and contents of carotenoids lead to the color ranging from white to yellow and red. The watermelon flesh color is a vital appearance quality and closely related to consumers’ preferences. The accumulation patterns of seven carotenoids in 4 flesh-colored watermelon during fruit development were detected by Lv et al. [49]. Eleven carotenoids and six isomers in red and yellow flesh-colored mature watermelon were detected by Liu et al. [14]. Twelve carotenoids in red, orange, and yellow flesh-colored mature watermelon fruits were measured by Fang et al. [44]. In this study, we have measured 13 carotenoids during fruit development in five flesh-colored watermelon genotypes (all are cultivated genotypes) using LC-MS/MS. The fruit flesh was white at the early developmental stages, then changed to various colors at the later stages due to the difference in pigments accumulation. Lycopene was the main pigment in red- and pink-fleshed genotypes consistent with the previous reports [44, 49]. In this study we also observed γ-Carotene, zeaxanthin, α-carotene were accumulated in the red fruit. The orange flesh color was largely determined by the content of β-carotene as in previous report [14]. Moreover, in the current study, we found that orange flesh also possesses the highest apocarotenal, β-cryptoxanthin, lutein, zeaxanthin, α-carotene, and neoxanthin levels. The orange-fleshed fruit may become a new health-care consumption type because of all kinds of carotenoids and higher total carotenoids contents. The previous study reported that violaxanthin, lutein, or neoxanthin are the dominant carotenoids in yellow flesh [14, 17, 49]. However, the antheraxanthin, zeaxanthin, and β-cryptoxanthin were also accumulated in yellow-fleshed fruits used here. The violaxanthin and lutein were accumulated in trace amounts in the white flesh [49], the antheraxanthin was observed in white flesh fruits as a new discovery here. In addition, we also determined the accumulation pattern of phytofluene and phytoene in five genotypes during fruit development, they are important upstream metabolites of carotenoid biosynthesis pathway. The lycopene was observed in the orange-fleshed fruits, it may exist as an intermediate metabolite for β-carotene. The α-carotene availability may partially explain the lutein content in the watermelon. Apocarotenal was specifically accumulated in orange-fleshed fruits, which may produce a unique flavor for this genotype [2]. To summarize, we detected 13 carotenoids in five genotypes and the most comprehensive accumulation patterns of carotenoids during fruit development in different colored watermelons were obtained. As special phenotypic traits, the compositions and contents of carotenoids are the basis of molecular research.

### Regulation of carotenoid biosynthesis pathway in different flesh-colored watermelons

**Fig. 8:**
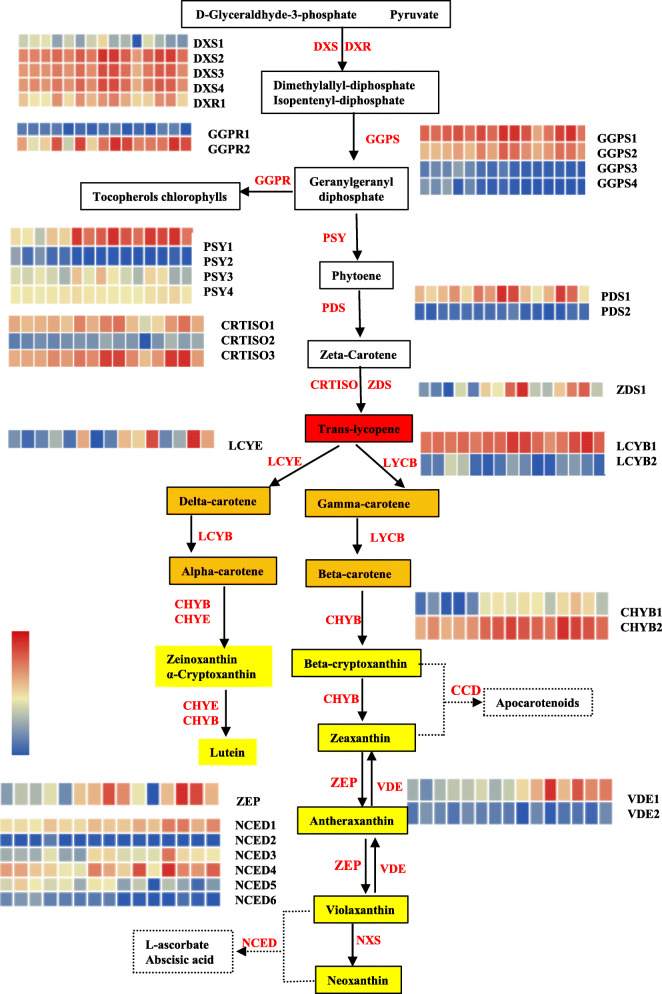
Expression profiles of genes involved in the carotenoid pathway of different flesh-colored watermelons. The FPKM of genes was listed in Additional file 1: Table S11. The heatmap cell from left to right represents R_10DAP, P_10 DAP, O_10 DAP, Y_10 DAP, W_10 DAP, R_20 DAP, P_20 DAP, O_20 DAP, Y_20 DAP, W_20 DAP, R_34 DAP, P_34 DAP, O_34 DAP, Y_34 DAP, and W_34 DAP. The colored cell represents the normalized gene expression levels according to the color scale. Metabolites background was colored according to their compound colors. Enzymes were presented in red font. DXS, 1-deoxy-D-xylulose-5-phosphate synthase; DXR, 1-deoxy-D-xylulose-5-phosphate reductoisomerase; GGPS, geranylgeranyl diphosphate synthase; GGPR, Geranylgeranyl diphosphate reductase; PSY, phytoene synthase; PDS, phytoene desaturase; ZDS, ζ-carotene desaturase; CRTISO, carotenoid isomerase; LCYE, lycopene ε-cyclase; LCYB, lycopene β-cyclase; CHYB, β-carotene hydroxylase; ZEP, zeaxanthin epoxidase; VDE, Violaxanthin de-epoxidase; NXS, neoxanthin synthase; NCED, 9-cis-epoxycarotenoid dioxygenase

The flesh color is due to the accumulation of pigments which are regulated and controlled by a complicated network consisting of a series of biosynthesis-, degradation-, and stable storage-related genes. To determine the potential regulatory networks underlying pigment contents in watermelon flesh, we performed comparative transcriptome analysis combined with WGCNA to identify hub genes highly correlated with carotenoid accumulation. In our study, 44 carotenoid pathway genes were differentially expressed in different samples of 5 genotypes (Additional file 1: Table S11), and most of them were assigned into carotenoids pathways (Fig. 8). The phytoene synthase (*Cla009122*), a rate-limiting enzyme in carotenoid biosynthesis flux, was highly expressed at the later developmental stages and served the carotenoid accumulation in fruits (Fig. 8). Expression of *Cla009122* gene was proportional to phytofluene content (r = 0.74), but not to total carotenoids content (r = 0.39). Perhaps the phytoene synthase is a key factor in carotenoid synthesis but not a determinant factor for every downstream metabolite accumulation. Lycopene beta-cyclase is an important branch point of the carotenoid synthesis pathway (Fig. 8). The expressions levels of lycopene beta-cyclase (*Cla005011*) was positively correlated with the contents of neoxanthin (r = 0.81), antheraxanthin (r = 0.75), and violaxanthin (r = 0.75), but weakly correlated with the content of lycopene (r = − 0.44), indicating that *Cla005011* gene expression level was not the main reason of lycopene accumulation. Actually, the lycopene content is related to the lycopene β-cyclase protein expression level [23]. Beta-carotene hydroxylase (*Cla006149*) was highly expressed in the orange and yellow flesh at later development stages, may contribute to the xanthophylls synthesis. But the highest gene expression level of beta-carotene hydroxylase in mature pink fruits indicating a more complex regulatory mechanism in this genotype (Additional file 1: Table S11, Fig. 6). The orange gene, *BoOr* and *CmOr*, encodes a plastidial DNA J cysteine-rich domain-containing protein and is an important regulator for carotenoid biosynthesis in cauliflower [50] and orange melon fruit [51]. *Cla018406* was homologous to the *BoOr* (E-value: 5e-120; Identity: 61.44%) and *CmOr* (E-value: 3e-130; Identity: 65.00%) and its gene expression pattern was related to the orange flesh and β-Carotene content. Thus, we considered *Cla018406* to be a strong candidate gene for orange flesh (Additional file 2: Fig. S9, Additional file 1: Table S11) on chromosome 4, different from the previously identified QTL associated with β-carotene accumulation on chromosome 1 in watermelon [21].

Many transcription factors were involved in the regulation of carotenoid metabolism [2]. We found some differentially expressed transcription factor genes in this study (Additional file 2: Fig. S10a). *SlBBX20* (zinc-finger transcription factor) is a positive regulator of carotenoid accumulation in tomato [52]. Here we identified the expression level of *Cla007686* (a zinc finger CCCH domain-containing protein) was associated with the lycopene content (r = 0.81) as described in the results. To further confirm this result, the relative gene expression levels of *Cla007686* and lycopene contents in 53 additional watermelon accessions were measured (Additional file 2: Fig. S11a, Additional file 1: Table S12), and a positive correlation between the expression level and lycopene content was obtained (r = 0.77) (Additional file 2: Fig. S11b). Then we suspected that *Cla007686* may be a key regulator for lycopene accumulation. Previous studies showed that transcription factor Golden2-Like2 (MYB) was involved in chloroplast biogenesis in Arabidopsis and tomato [38, 39]. Consistent with this, we found *Cla010265* and *Cla020369* (*GLK2* TFs) were related to the content of lycopene. The R2R3-MYB protein family act as a regulatory function in the carotenoid pathway in tomato [53]. Here, the gene expression of *Cla009263* and *Cla017995* (*R2R3-MYB*) were differentially expressed in 5 genotypes at 34 DAP (Additional file 2: Fig. S10a, Additional file 1: Table S11) and related to the phytoene content. The transcription factor *SlMADS1* and *CsMADS6* are important in the tomato ripening [40] and citrus fruit coloration [41], their homologous gene (*Cla000691* and *Cla010815*, *Cla009725* and *Cla019630*) were identified in this study. Maybe these transcription factors are the regulators to control the color formation of watermelon. Besides the potential transcription factors, we identified 26 DEGs related to chlorophyll biosynthesis (Additional file 2: Fig. S10b, Additional file 1: Table S11). Moreover, plastid is where carotenoids are synthesized and stored, plastid development is closely related to the accumulation of carotenoids. 22 DEGs were annotated to be involved in the plastid biogenesis (Additional file 2: Fig. S10c, Additional file 1: Table S11). The genes related to chlorophyll biosynthesis and plastid development may indirectly regulate the carotenoid pathway in watermelon. Carotenoid synthesis is a very complex process and varies with different genotypes. Mining and speculating of structural genes or transcription factors is the first step to elucidate the molecular mechanism of carotenoid accumulation.

Considering that molecular mechanisms underlying flesh color formation are still not well understood, the candidate genes provided in this study can be further verified by the molecular biology approach. The results will help further to understand the specific molecular mechanism of watermelon color formation.

## Conclusions

In this study, we performed comparative transcriptomics at different developmental stages among five cultivated watermelons with different flesh colors to understand the carotenoid accumulation patterns and regulatory mechanisms. The carotenoids contents in red- and orange-fleshed fruits were higher than that in pink-, yellow- and white-fleshed watermelon at mature stage. Through comparative transcriptome analysis, cluster analysis, GO term analysis, KEGG analysis, and WGCNA analysis, a number of candidate genes with respect to fruit development and color formation were reported here. The WGCNA is a useful method for identifying trait-specific modules and hub genes. We speculate *Cla018406* (chaperone protein dnaJ-like protein), *Cla007686* (a zinc finger CCCH domain-containing protein), *Cla003760* (membrane protein) and *Cla021635* (photosystem I reaction center subunit II) were candidate genes for orange, red, and yellow flesh, respectively. Further work on gene function validation is required to have deep insights of the genetic and molecular mechanisms underlying watermelon fruit coloration.

## Methods

### Plant materials and sampling

The seeds of 58 watermelon accessions were provided by the polyploidy watermelon research group (Zhengzhou, China), Zhengzhou Fruit Research Institute, Chinese Academy of Agricultural Sciences. The orange-fleshed inbred Qitouhuang, yellow-fleshed inbred Xihua, red-fleshed inbred Zhengzhou No. 3, pink-fleshed inbred Hualing, and the white-fleshed inbred Bingtangcui (Fig. 1) were used for different developmental stages analysis. Watermelon seeds were sown in pots (filled with nutritional media) in a greenhouse in April 2018. One-month-old watermelon seedlings were transplanted in the open field at the Xinxiang experimental farm (Xinxiang, Henan, China), with spacing as 30 cm between plants and 150 cm between rows. The plants were separated by genotype and replication. The field management followed common horticultural practices (fertilization, irrigation, pathogen prevention, and pest control) for open-field watermelon growing.

Flowers were hand-pollinated and tagged to record the number of days after pollination (DAP). Flesh samples were collected from uniform injury-free watermelon fruits at three critical development stages (10, 20, and 34 DAP). These samples were immediately frozen in liquid nitrogen and stored at − 80 °C until use. The pooled sample from three fruits was used as one biological replicate, three individual biological replicates for each treatment. Approximately 10 g and 50 g of flesh samples were collected for RNA-seq analysis and carotenoid profiles determination, respectively.

### Phenotyping

The fruits were picked, cut open longitudinally and visually scored for flesh color first. Images were taken from all fruits. CIE color space values (L*, a*, and b*) were measured on each fruit section using a Chroma-meter Konica-Minolta CR-400 (Japan). The color saturation was calculated by formula, Chroma (C) = [(a*)^2^ + (b*)^2^]^1/2^.

### Quantitation of carotenoids

The carotenoid extraction and measurement was performed as previously described [54]. The lyophilized flesh powder was extracted using hexane- acetone- ethanol (volumic ratio, 2:1:1) containing 0.01% butyl hydroxytoluene (BHT). The extracted samples were measured using a UPLC-APCI-MS/MS system (API 6500 Q TRAP). The measurement conditions and APCI source operation parameters were the same as previously described [54]. The chemical standards purchased from Sigma-Aldrich company (USA). The relative contents of each sample were corresponding to the spectral peak intensity values. The absolute content was calculated using linear equations of standard curves.

### RNA extraction and sequencing

For different watermelon flesh samples, total RNA was extracted using Plant Total RNA Purification Kit (GeneMark, Beijing, China) following the manufacturer’s instructions. The RNA degradation and contamination were monitored on 1% agarose gels. The RNA purity, concentration, and integrity were checked using the NanoPhotometer® spectrophotometer (IMPLEN, CA, USA), Qubit® RNA Assay Kit in Qubit® 2.0 Fluorometer (Life Technologies, CA, USA), and the RNA Nano 6000 Assay Kit of the Bioanalyzer 2100 system (Agilent Technologies, CA, USA), respectively.

A total amount of 5 μg total RNA of each sample was used. Sequencing libraries were generated according to Kit for Illumina. The clustering of the samples was performed on a cBot Cluster Generation System using TruSeq PE Cluster Kit v3-cBot-HS (Illumia). The library preparations were sequenced on an Illumina Hiseq platform and 125 bp/150 bp paired-end reads were generated. The high-quality data (clean reads) were obtained by removing reads containing adapter, reads containing ploy-N and low-quality reads from raw data. At the same time, Q20, Q30, and GC content of the clean data were calculated. The watermelon reference genome (97,103 V1) was downloaded from the website (http://cucurbitgenomics.org/organism/1). Paired-end clean reads were aligned to the reference genome using Hisat2 v2.0.4.

### Quantification of gene expression level

HTSeq v0.9.1 was used to count the reads numbers mapped to each gene and then FPKM of each gene was calculated based on the length of the gene and reads count mapped to this gene [28].

### Differential expression analysis

Differential expression analysis was performed using the DESeq R package (1.18.0). Genes with an adjusted *P*-value < 0.05 found by DESeq were assigned as differentially expressed.

### GO term and KEGG enrichment analysis of differentially expressed genes

Gene Ontology (GO) enrichment analysis of differentially expressed genes was implemented by the GOseq R package, in which gene length bias was corrected. GO terms with corrected *P* value less than 0.05 were considered significantly enriched by differential expressed genes. We used KOBAS software to test the statistical enrichment of differential expression genes in KEGG pathways.

### Co-expression networks analysis

Co-expression networks analysis was performed using R package WGCNA [55] and visualized using Cytoscape software [56], based on 16,781 normalized FPKM values and the trait data representing carotenoid levels in different samples. The hub genes in each module were analyzed using cytohubba.

### Validation of DEGs expression by qRT-PCR

The first-strand cDNA was synthesized from 1 μg RNA using a Prime ScriptTM RT reagent kit with gDNA Eraser (TaKaRa, Kusatsu, Shiga, Japan) based on the manufacturer’s protocol. The cDNA was synthesized from 1 μg of total RNA with PrimeScript™ RT reagent Kit with gDNA Eraser following the manufacturer’s instructions (Takara, Japan). For quantitative reverse transcription polymerase chain reaction (qRT-PCR), relative gene expression levels of the target gene were measured using a Roche LightCycler480 RT-PCR system (Roche, Swiss). The SYBR Green real-time PCR mix was added to the reaction system according to the manufacturer’s instructions. The primers were designed using Primer premier 6 based on Cucurbit Genomics Database (http://cucurbitgenomics.org/) and listed in Additional file 1: Table S13. All genes were run in triplicate from the three biological replicates. The raw data of qRT-PCR were analyzed using LCS 480 software 1.5.0.39 (Roche, Swiss) and the relative expression was determined by using the 2^−ΔΔCT^ method. The watermelon *ClACTIN* genes were used as internal control [57].

### Statistical analysis

Statistical analysis for color parameters and carotenoids contents were conducted using SPSS 19.0 and according to Tukey’s post-hoc test.

## Supplementary Information

**Additional file 1 Table S1.** Estimation of color coordinates values in the watermelon fruits of different development stages. **Table S2.** The contents of carotenoids compounds (ug/g). **Table S3.** An overview of the RNA-Seq data. **Table S4.** Numbers of detected transcripts in each sample. **Table S5.** Pearson correlation coefficient list between samples. **Table S6.** Numbers of differentially expressed genes (DEGs) in each Pairwise comparison. **Table S7.** FPKM of genes listed in this study. **Table S8.** Gene list of each subcluster. **Table S9.** Gene list of modules. **Table S10.** Hub gene in modules calculated using cytohubba. **Table S11.** The genes related to carotenoid biosynthesis. **Table S12.** The lycopene content and the gene expression of *Cla007686* in 53 watermelon accessions. **Table S13.** Primers used for the quantitative real-time PCR analysis.

**Additional file 2 Fig. S1.** PCA plots for all samples using log-transformation data of the relative content of carotenoid metabolites. **Fig. S2.** The expression of selected key DEGs listed in this study. **Fig. S3.** Go term analysis of Subcluster 1–6. **Fig. S4.** (a) Sample dendrogram and module trait heatmap at each developmental stage. (b) The parameter, soft threshold, determination for module construction. **Fig. S5.** (a) Genes cluster dendrogram (hierarchical clustering tree) of the transcriptome. (b) Network heatmap of selected genes. **Fig. S6.** Go term and KEGG analysis of genes in yellow module. **Fig. S7.** Heat cluster analysis of (a) yellow module, (b) darkred module, and (c) purple module. **Fig. S8.** The heatmap of hub genes. **Fig. S9.** Validation of selected DEGs expression by qRT-PCR. **Fig. S10.** The heatmap of (a) key transcription factor genes, (b) chlorophyll biosynthesis genes, and (c) plastid biogenesis genes. **Fig. S11.** (a) The relative gene expression levels of *Cla007686* and relative lycopene contents in 53 watermelon accessions. (b) The correlation between lycopene content and *Cla007686* gene mRNA levels.

**Additional file 3 Dataset 1.** DEGs at 10 DAP between the genotypes.

**Additional file 4 Dataset 2.** DEGs at 20 DAP between the genotypes.

**Additional file 5 Dataset 3.** DEGs at 34 DAP between the genotypes.

**Additional file 6 Dataset 4.** DEGs between different stages in each genotype.

**Additional file 7 Dataset 5.** Go and KEGG analysis of genes in six subclusters.

## Data Availability

The transcriptome raw reads have been deposited as a BioProject under accession: PRJNA644468. The materials are available from the corresponding author on reasonable request after the publication of the work.

## Abbreviations

DAP: days after pollination
R: red
P: pink
O: orange
Y: yellow
W: white
DEGs: differentially expressed genes
TFs: transcription factors
WGCNA: weighted gene coexpression network analysis
C: Chroma
PCA: Principal Component Analysis
FPKM: Reads Per Kilobase per Million mapped reads
qRT-PCR: Quantitative real-time polymerase chain reaction
GO: Gene Ontology
KEGG: Kyoto Encyclopedia of Genes and Genomes
TCA cycle: tricarboxylic acid cycle
cc: correlation coefficient
BHT: butyl hydroxytoluene
DXS: 1-deoxy-D-xylulose-5-phosphate synthase
DXR: 1-deoxy-D-xylulose-5-phosphate reductoisomerase
GGPS: geranylgeranyl diphosphate synthase
GGPR: Geranylgeranyl diphosphate reductase
PSY: phytoene synthase
PDS: phytoene desaturase
ZDS: ζ-carotene desaturase
CRTISO: carotenoid isomerase
LCYE: lycopene ε-cyclase
LCYB: lycopene β-cyclase
CHYB: β-carotene hydroxylase
ZEP: zeaxanthin epoxidase
VDE: Violaxanthin de-epoxidase
NXS: neoxanthin synthase
NCED: 9-cis-epoxycarotenoid dioxygenase
