# Supplementary material for: Transcriptome regulation of carotenoids in five flesh-colored watermelons (Citrullus lanatus)
Source: BMC Plant Biol. 2021 Apr 28;21:203. doi: 10.1186/s12870-021-02965-z (PMC8082968; doi:10.1186/s12870-021-02965-z)
Supplement: Supplementary file 2 — Additional file 2 Fig. S1. PCA plots for all samples using log-transformation data of the relative content of carotenoid metabolites. Fig. S2. The expression of selected key DEGs listed in this study. Fig. S3. Go term analysis of Subcluster 1–6. Fig. S4. (a) Sample dendrogram and module trait heatmap at each developmental stage. (b) The parameter, soft threshold, determination for module construction. Fig. S5. (a) Genes cluster dendrogram (hierarchical clustering tree) of the transcriptome. (b) Network heatmap of selected genes. Fig. S6. Go term and KEGG analysis of genes in yellow module. Fig. S7. Heat cluster analysis of (a) yellow module, (b) darkred module, and (c) purple module. Fig. S8. The heatmap of hub genes. Fig. S9. Validation of selected DEGs expression by qRT-PCR. Fig. S10. The heatmap of (a) key transcription factor genes, (b) chlorophyll biosynthesis genes, and (c) plastid biogenesis genes. Fig. S11. (a) The relative gene expression levels of Cla007686 and relative lycopene contents in 53 watermelon accessions. (b) The correlation between lycopene content and Cla007686 gene mRNA levels. [file 12870_2021_2965_MOESM2_ESM.pdf]

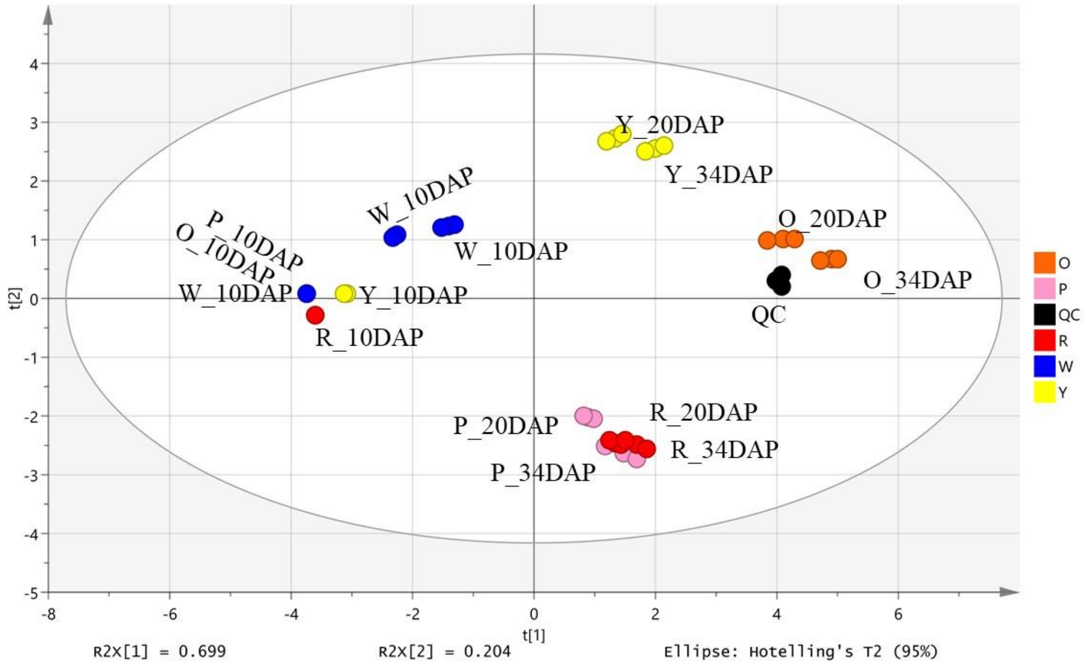

Fig. S1 PCA plots for all samples using log-transformation data of the relative content of carotenoid metabolites. T [1] and T [2] explain 69.9% and 20.4%, respectively. Each variety is represented by dots in different colors. A clear distinction between flesh color can be seen in the time-course. R, P, O, Y, and W represents the red-, pink-, orange-, yellow-, and white-fleshed genotypes, respectively; DAP, days after pollination; QC, quality control samples.

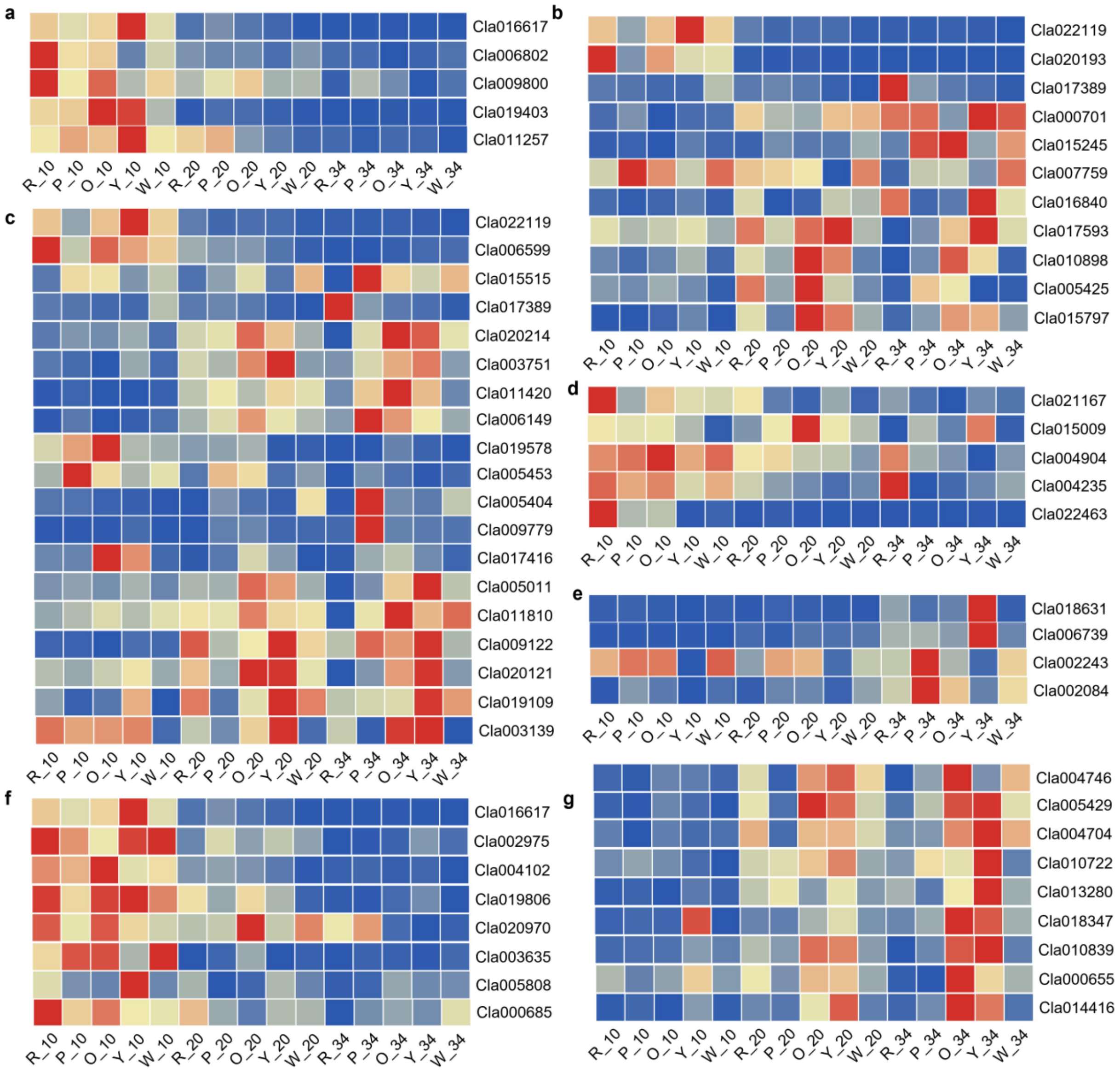

Fig. S2 The expression of selected key DEGs listed in this study. Each gene was analyzed separately. Red represents the high expression level, and blue represents the low expression level. R, P, O, Y, and W represents the red-, pink-, orange-, yellow-, and white-fleshed genotypes, respectively. 10, 20, and 34 represents the different developmental stages.

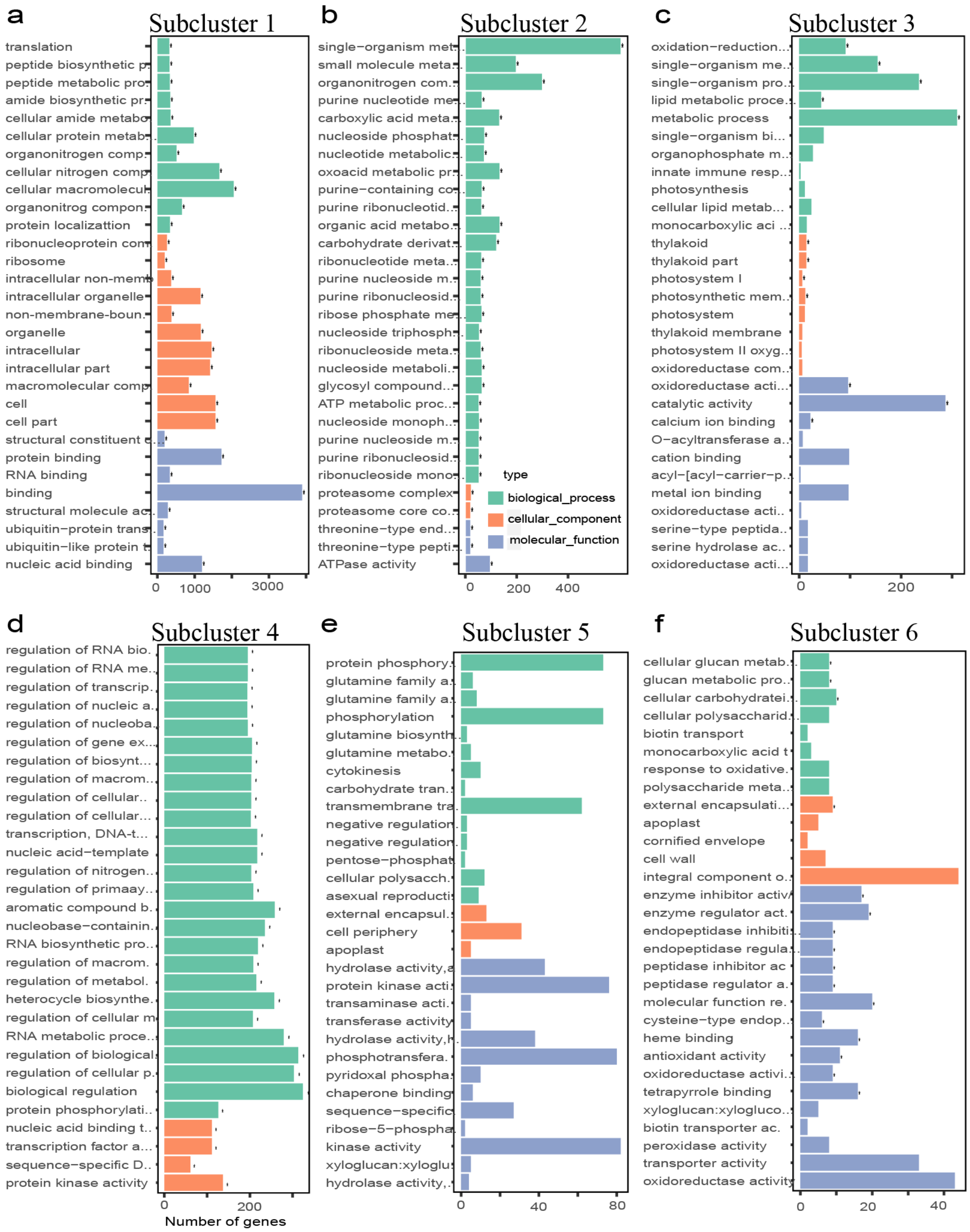

Fig. S3 Go term analysis of Subcluster1-6.

The asterisk on the column indicates significant enrichment, the figure related to supplementary dataset 5.

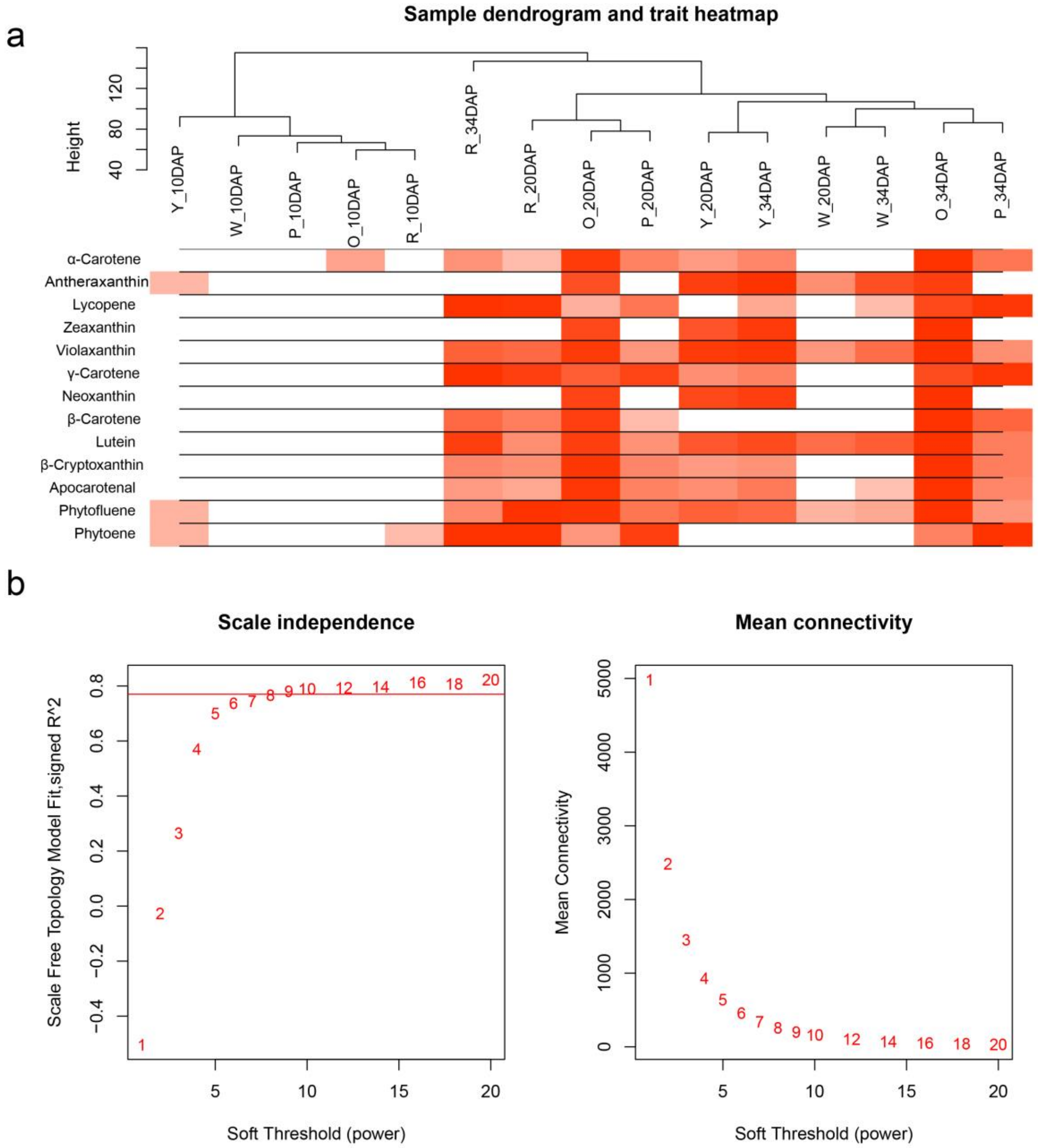

Fig. S4 (a) Sample dendrogram and module trait heatmap at each developmental stage. (b) The parameter, soft threshold, determination for module construction. The best value is 7.7 for this dataset.

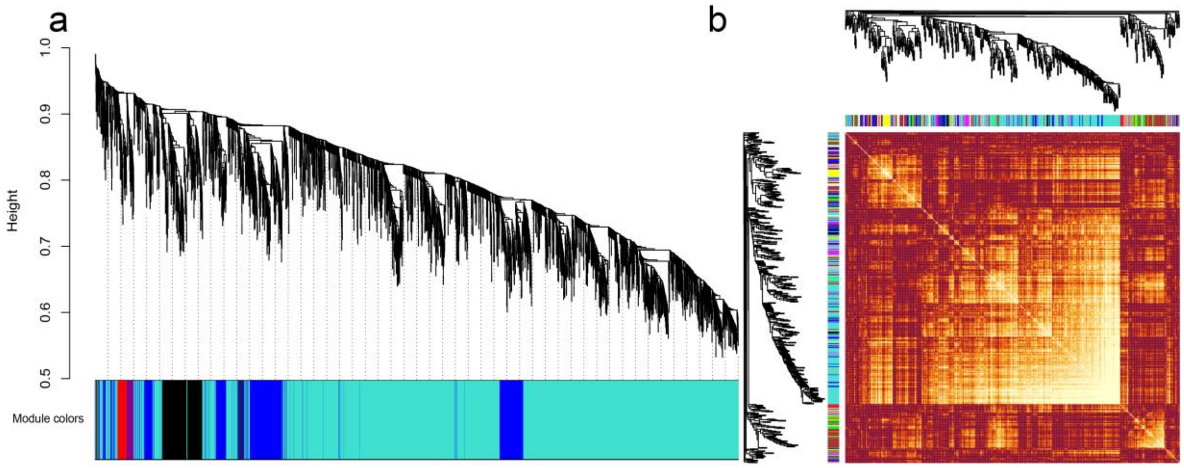

Fig. S5 (a) Genes cluster dendrogram (hierarchical clustering tree) of the transcriptome. Each leaf in the tree represents one gene. 40 modules were built based on gene expression level (labeled with different colors). (b) Network heatmap of selected genes.

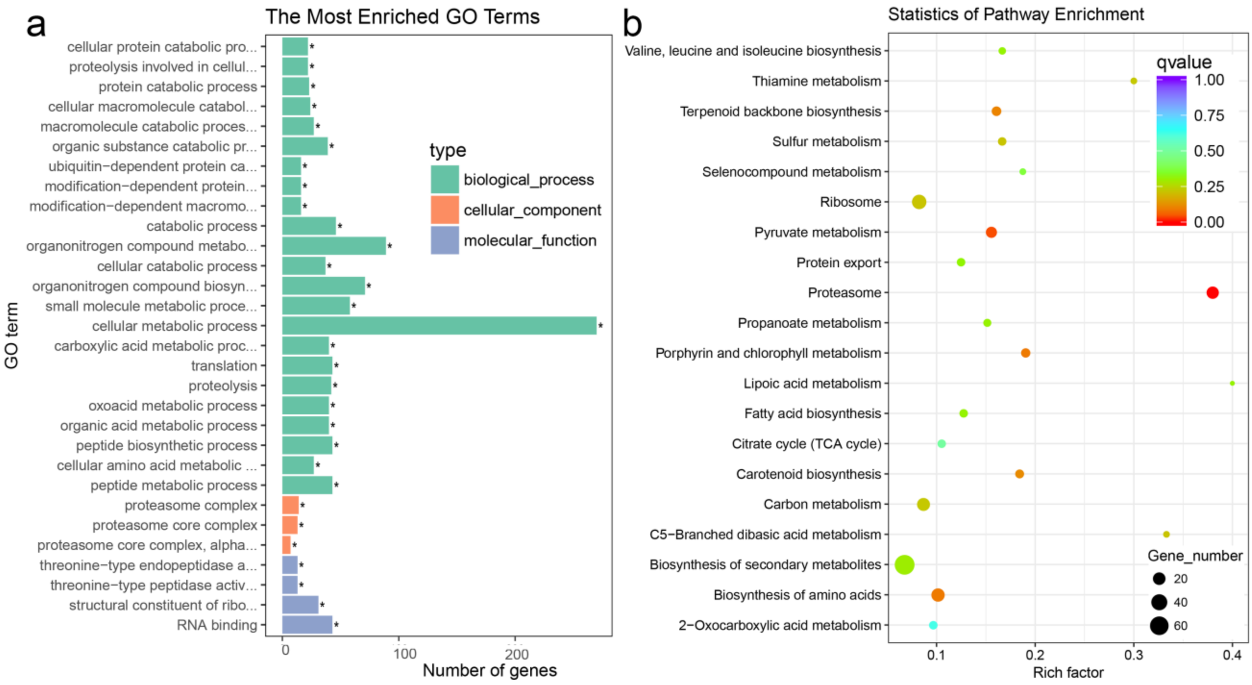

Fig. S6 Go term and KEGG analysis of genes in yellow module

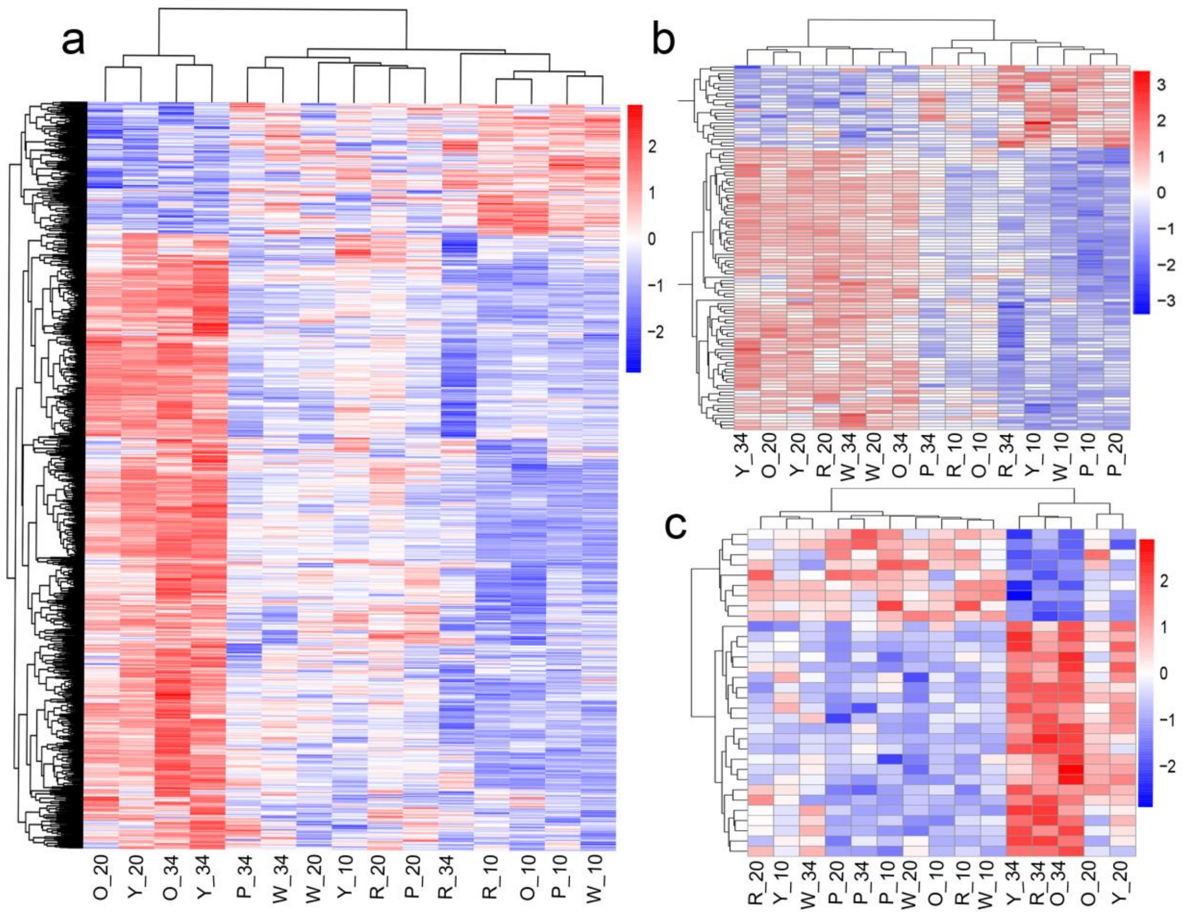

Fig. S7 Heat cluster analysis of (a) yellow module, (b) darkred module, and (c) purple module. R, P, O, Y, and W represents the red-, pink-, orange-, yellow-, and white-fleshed genotypes, respectively. 10, 20, and 34 represents the different developmental stages.

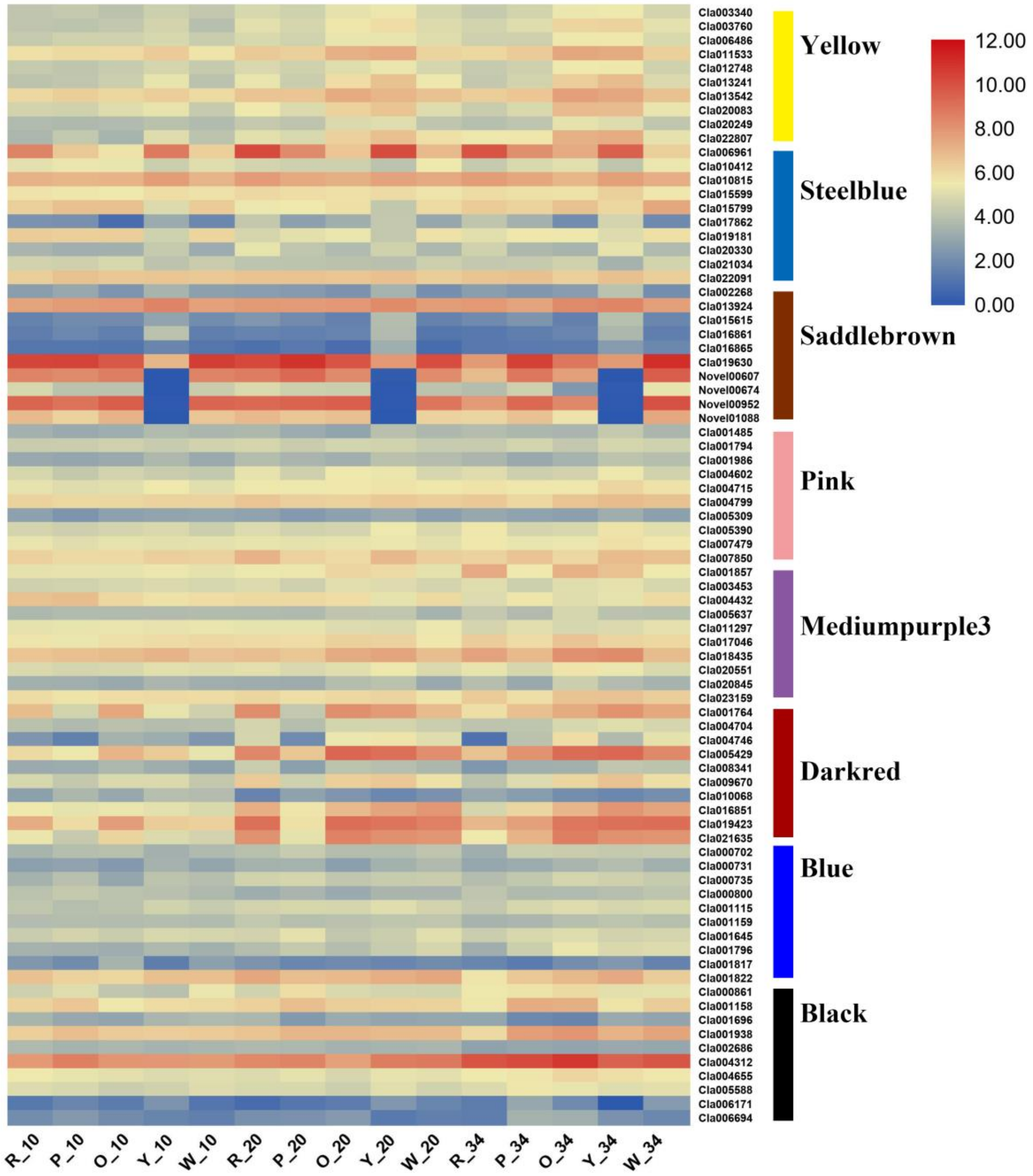

Fig. S8 The heatmap of hub genes. The log10 (FPKM+1) value was normalized and transformed. Red represents the high expression level, and blue represents the low expression level. R, P, O, Y, and W represents the red-, pink-, orange-, yellow-, and white-fleshed genotypes, respectively. 10, 20, and 34 represents the different developmental stages.

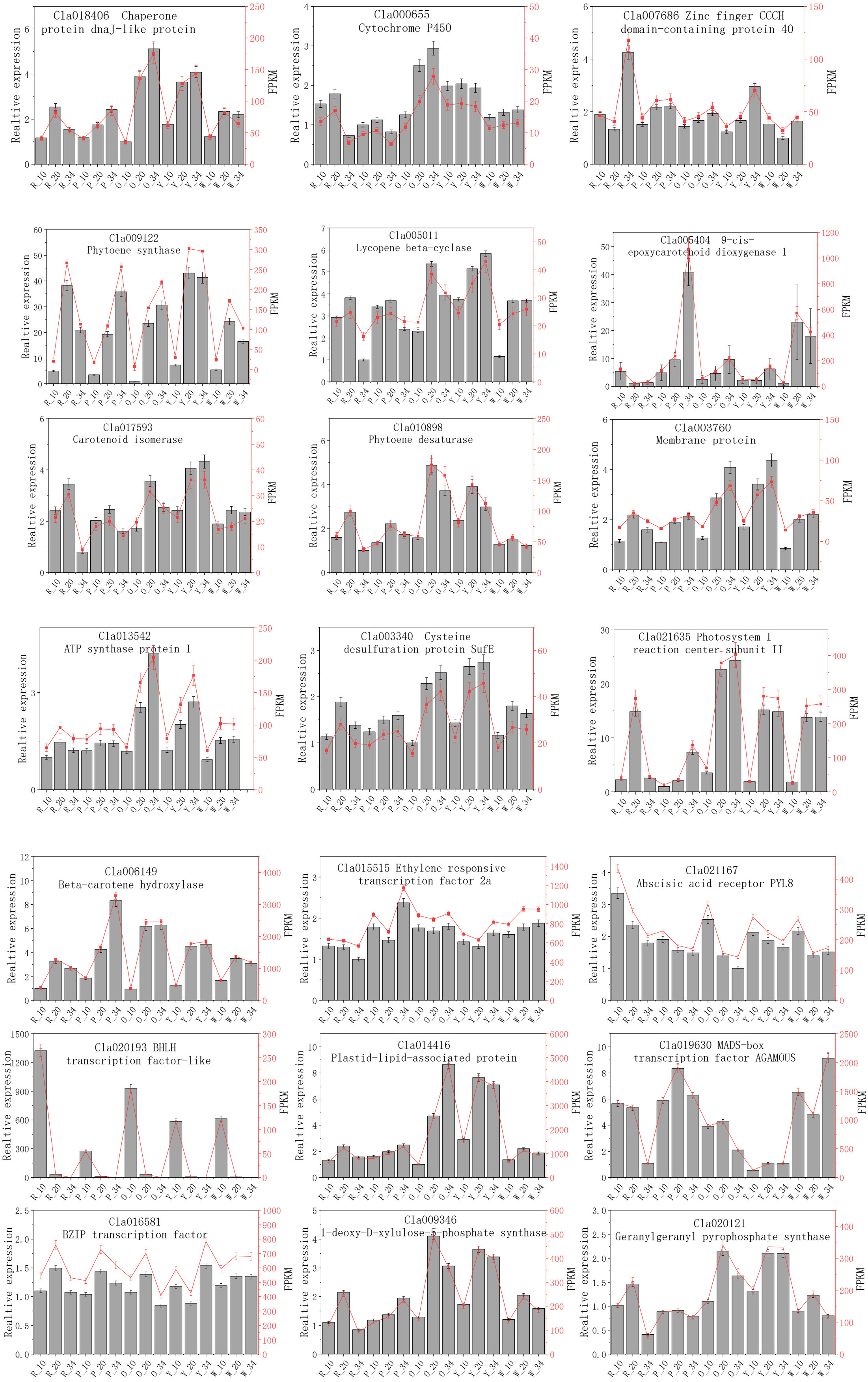

Fig. S9 Validation of selected DEGs expression by qRT-PCR.

Transcript abundance changes were detected by FPKM values according to RNA-Seq(left colume). Red lines and column represent the FPKM values and RT-qPCR expression values, respectively. Values are reported as means ± SEs (n ≥ 3). Three biological replications were included in the analysis. R, P, O, Y, and W represents the red-, pink-, orange-, yellow-, and white-fleshed genotypes, respectively. 10, 20, and 34 represents the different developmental stages.

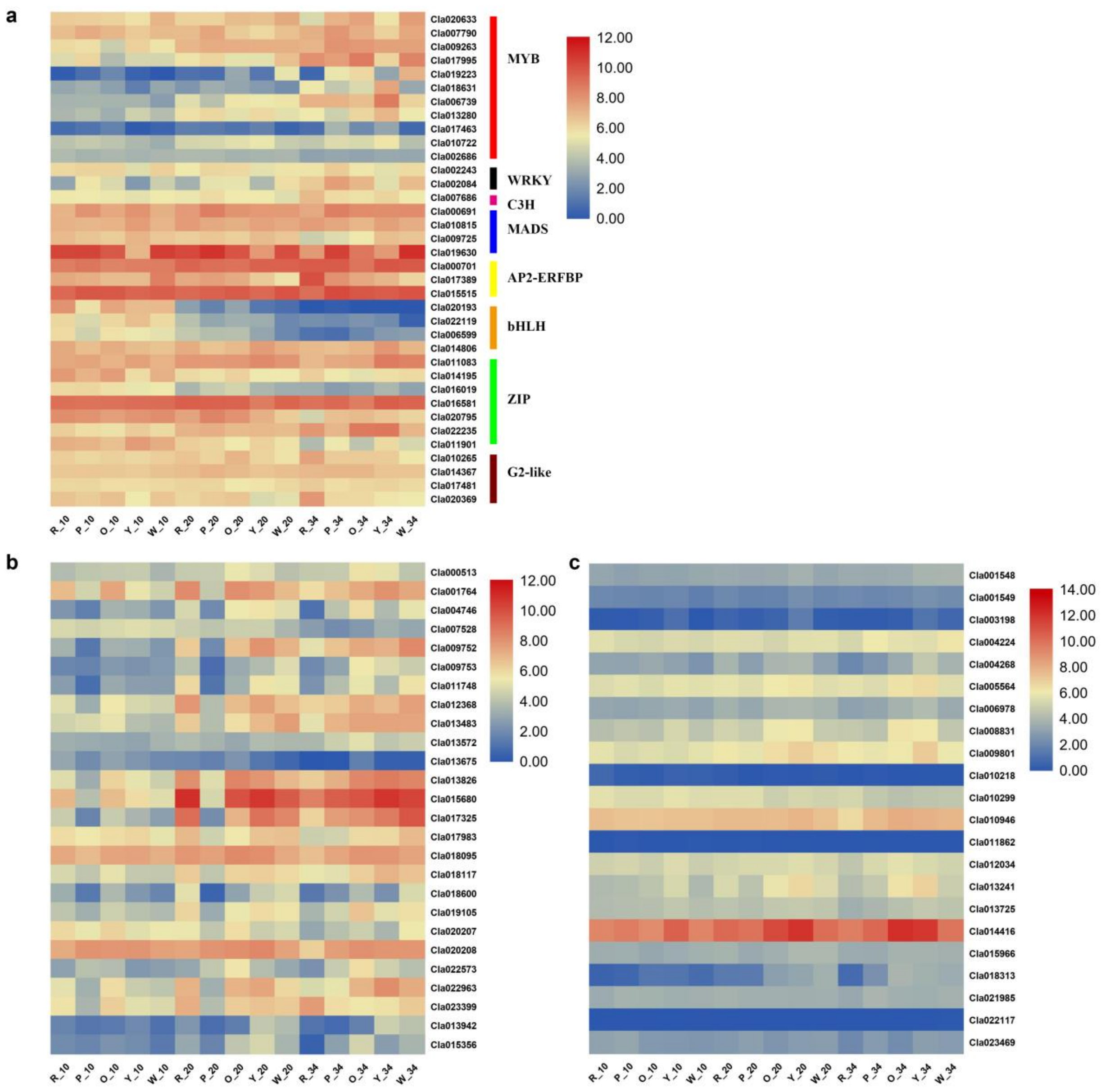

Fig. S10 The heatmap of (a) key transcription factor genes, (b) chlorophyll biosynthesis genes, and (c) plastid biogenesis genes. The log10 (FPKM+1) value was normalized and transformed. Red represents the high expression level, and blue represents the low expression level. R, P, O,

Y, and W represents the red-, pink-, orange-, yellow-, and white-fleshed genotypes, respectively. 10, 20, and 34 represents the different developmental stages.

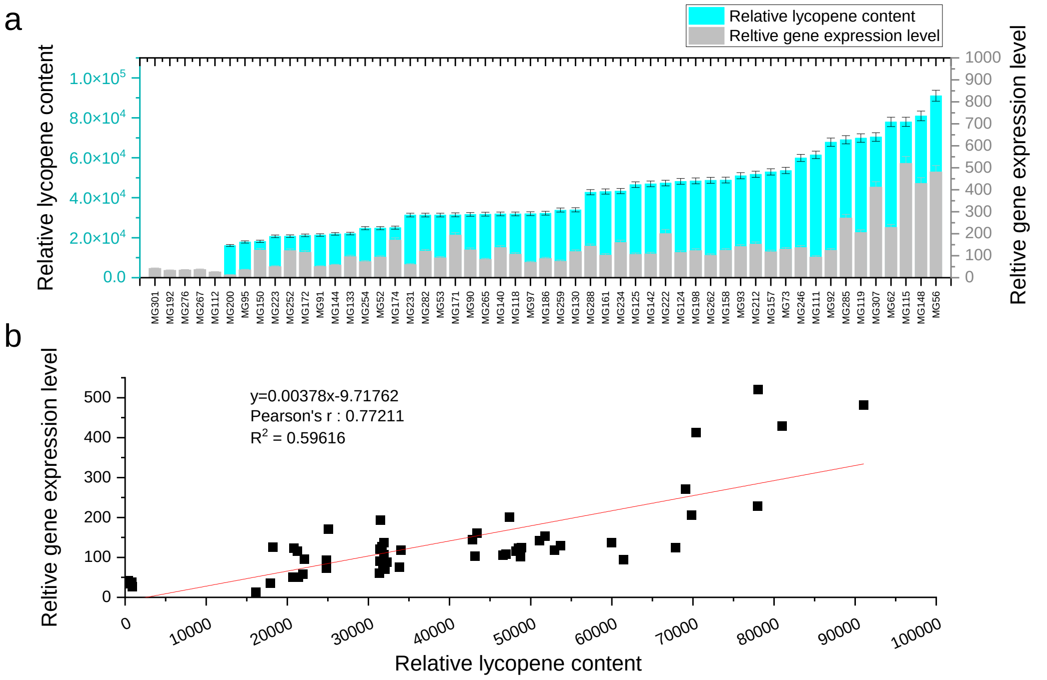

Fig. S11 (a) The relative gene expression levels of *Cla007686* and relative lycopene contents in 53 watermelon accessions. (b) The correlation between lycopene content and *Cla007686* gene mRNA realtive levels. The relative gene expression levels determined by qRT-PCR in mature fruits. The relative lycopene contents were represented by the peak area of high performence liquid chromatography system. Values are reported as means ± SEs (n = 3).
